# Phenotypic Subtyping and Re-Analysis of Existing Methylation Data from Autistic Probands in Simplex Families Reveal ASD Subtype-Associated Differentially Methylated Genes and Biological Functions

**DOI:** 10.3390/ijms21186877

**Published:** 2020-09-19

**Authors:** Elizabeth C. Lee, Valerie W. Hu

**Affiliations:** Department of Biochemistry and Molecular Medicine, The George Washington University, School of Medicine and Health Sciences, Washington, DC 20037, USA; elee171@jhu.edu

**Keywords:** phenotypic subgroups stratified by ASD severity, simplex families, DNA methylation, subgroup-associated genes and biological functions

## Abstract

Autism spectrum disorder (ASD) describes a group of neurodevelopmental disorders with core deficits in social communication and manifestation of restricted, repetitive, and stereotyped behaviors. Despite the core symptomatology, ASD is extremely heterogeneous with respect to the severity of symptoms and behaviors. This heterogeneity presents an inherent challenge to all large-scale genome-wide omics analyses. In the present study, we address this heterogeneity by stratifying ASD probands from simplex families according to the severity of behavioral scores on the Autism Diagnostic Interview-Revised diagnostic instrument, followed by re-analysis of existing DNA methylation data from individuals in three ASD subphenotypes in comparison to that of their respective unaffected siblings. We demonstrate that subphenotyping of cases enables the identification of over 1.6 times the number of statistically significant differentially methylated regions (DMR) and DMR-associated genes (DAGs) between cases and controls, compared to that identified when all cases are combined. Our analyses also reveal ASD-related neurological functions and comorbidities that are enriched among DAGs in each phenotypic subgroup but not in the combined case group. Moreover, relational gene networks constructed with the DAGs reveal signaling pathways associated with specific functions and comorbidities. In addition, a network comprised of DAGs shared among all ASD subgroups and the combined case group is enriched in genes involved in inflammatory responses, suggesting that neuroinflammation may be a common theme underlying core features of ASD. These findings demonstrate the value of phenotype definition in methylomic analyses of ASD and may aid in the development of subtype-directed diagnostics and therapeutics.

## 1. Introduction

Autism spectrum disorder (ASD) is a complex neurodevelopmental disorder characterized by impaired social communication and repetitive behaviors [1]. Tremendous phenotypic and symptomatic heterogeneity exists within the ASD population, thereby presenting a challenge to diagnosis and treatment. The wide range of clinical presentation in ASD is attributed to different underlying etiologies, which include both genetic and environmental influences. One area that bridges the genetics–environment gap is epigenetic variation, which has been proposed to play a role in ASD [2,3,4,5,6]. It has been shown that DNA methylation is dysregulated in ASD in multiple studies involving both peripheral and brain tissues, principally from individuals with ASD from the multiplex population [7,8,9,10,11,12,13,14,15]. However, published DNA methylation studies of ASD have produced inconsistent findings, including variable reporting of differentially methylated sites. This inconsistency may be explained not only by the different tissues used but also in part by the wide phenotypic heterogeneity intrinsic to ASD. 

Previous findings from our laboratory showed that reduction of ASD clinical heterogeneity by classifying patients into subphenotypes based on cluster analyses of severity scores from the Autism Diagnostic Interview-Revised (ADI-R) diagnostic instrument [16] results in increased ability to detect statistically significant subphenotype-specific transcriptomic as well as genetic differences, which were otherwise undetectable in an aggregate analysis of all individuals with ASD [17,18,19,20]. Based on these previous studies that demonstrate the value of subphenotyping in genome-wide omics analyses and the growing body of evidence implicating a link between ASD and epigenetic modification, we hypothesized that the stratification of individuals with ASD by phenotypic severity will result in the identification of subphenotype-dependent DNA methylation differences between cases and controls that achieve statistical significance. 

The present study involves re-analyzing existing Illumina HumanMethylation27K BeadChip data from lymphoblastoid cell lines (LCLs) derived from blood lymphocytes of 292 male ASD probands from the Simons Simplex Collection (SSC) after stratification into three distinct subgroups based on ADI-R symptom severity profiles (mild, intermediate, and severely language-impaired). The main goals of this research are to: (1) identify statistically significant differences in DNA methylation between cases and typically developing sibling controls for each of the three ASD subphenotypes, (2) examine the impact of decreasing phenotypic heterogeneity on the ability to detect statistically significant differentially methylated regions and associated genes (DAGs) by comparing results with and without subphenotyping, and (3) identify biological functions, signaling pathways, and disorders associated with DAGs from each subgroup analysis. 

## 2. Results and Discussion

### 2.1. DAGs Associated with ASD Subphenotypes and the Combined Case Group 

**Figure 1:**
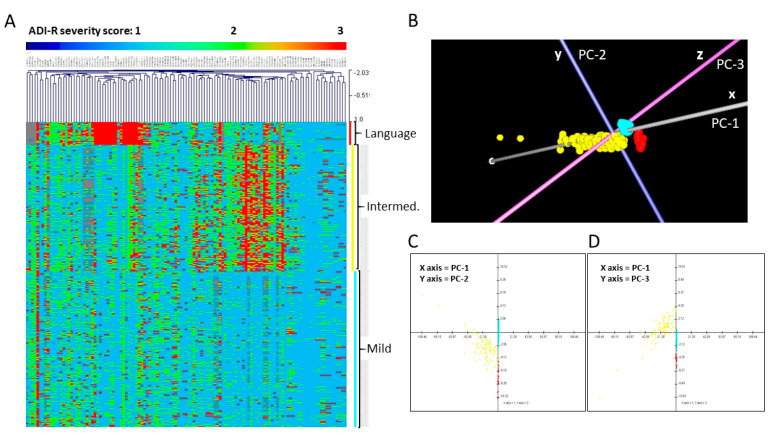
Separation of probands into three phenotypic subgroups based on cluster analyses of 123 scores on 63 Autism Diagnostic Interview-Revised (ADI-R) items for each individual. (**A**) Hierarchical clustering (HCL) analysis, (**B**–**D**) Principal components analysis (PCA) in 3-d (**B**) and 2-d projections showing PC-1 and PC-2 (C) and PC-1 and PC-3 (D). For the heatmap in (**A**), each row represents an individual, while each column represents a score from the ADI-R diagnostic. The range of severity scores (1–3) for each ADI-R item is represented in the color bar above the heatmap, with light blue indicating a score of 1, green-yellow indicating a score of 2, and red indicating a score of 3, which represents the most severe autism spectrum disorder (ASD) manifestation. The three ASD subgroups are identified by the vertical colored bars along the right side of the heatmap, with red indicating the severely language-impaired subgroup, yellow indicating the intermediate subgroup, and turquoise indicating the mild subgroup. This latter set of colors also applies to the subgroups shown in the PCA plots in which each point represents an individual. Note: In the heatmap (**A**), the large block of red columns associated with the severely language-impaired subgroup primarily corresponds to items involving spoken language on the ADI-R diagnostic.

Hierarchical clustering (HCL) and principal components analysis (PCA) using scores on the ADI-R diagnostic scoresheets from each of the probands were performed as previously described [16]. These cluster analyses confirmed that the 292 cases in this methylation study could be separated into three phenotypic subgroups based on their severity scores from the ADI-R. A heatmap depicting clinical severity across 123 scores on 63 ADI-R items for individuals in each subgroup is shown in Figure 1, together with PCA plots from the data reduction analysis confirming the separation of cases into three distinguishable subgroups according to integrated severity profiles. Notably, the first three principal components (represented by the x, y, and z-axes of the 3-d PCA plot) account for 85.72% of the variability among all probands based on the 123 ADI-R scores.

**Figure 2:**
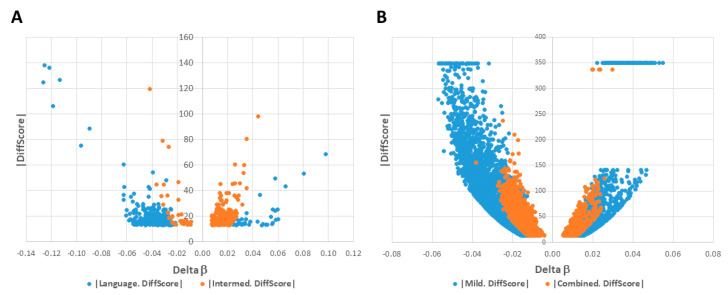
Volcano plots of significant differentially methylated regions and associated genes (DAGs) among subgroups and combined cases. |DiffScore| versus Delta β plots for significant DAGs in: (**A**) severely language-impaired (blue) and intermediate (red) subgroups; (**B**) mild subgroup (blue) and combined cases (red). Note: A |DiffScore| of 13 is roughly equivalent to a *p*-value of 0.05.

Using GenomeStudio Methylation Module software, CpG sites across the genome were identified that exhibited statistically significant differential methylation with False Discovery Rate (FDR)-adjusted *p*-values < 0.05. For the severely language-impaired subgroup (*n* = 22 cases and 22 controls), 266 unique DAGs were mapped to the CpGs (Table S1). The intermediate subgroup (*n* = 121 cases and 121 controls) exhibited 360 unique DAGs (Table S2), and the mild subgroup (*n* = 149 cases and 149 controls) exhibited 4073 unique DAGs (Table S3). Among the three ASD subgroups, a total of 4155 unique DAGs with FDR-adjusted *p*-values < 0.05 were identified, with some DAGs shared among the subgroups. The volcano plots for each subgroup illustrate distinct differences in the number, distribution, and methylation profiles of significant DAGs in each subgroup (Figure 2). For example, the majority of the DAGs in the severely language-impaired subgroup show reduced methylation (negative delta β values), while the majority of the DAGs in the intermediate subgroup show increased methylation (positive delta β values) (Figure 2A). Although the majority of DAGs in the mild subgroup show decreased methylation as observed in the severely language-impaired subgroup, the mild subgroup has a much greater number of significant DAGs (Figure 2B). In addition, while all the DAGs in the mild subgroup exhibit delta β values < |±0.05|, a fraction of the DAGs associated with the severely language-impaired subgroup exceeds these absolute delta β values, indicating larger methylation differences between cases and controls, which are also reflected by larger fold-change values (see Figure S1). These data suggest that the three ASD subgroups can be distinguished from each other by their differential DNA methylation profiles. 

To examine the impact of decreasing phenotypic heterogeneity on the ability to detect statistically significant DAGs, differential methylation analysis of the 27K BeadChip data using Illumina’s GenomeStudio Methylation Module was also performed without stratification into phenotypic subtypes, i.e., combined case group (*n* = 292 cases and 292 controls). Without subgrouping, a total of 2570 unique DAGs with FDR-adjusted *p*-values < 0.05 were identified in the combined case group (Table S4). The volcano plot of DAGs for the combined case group is shown in

**Figure 3:**
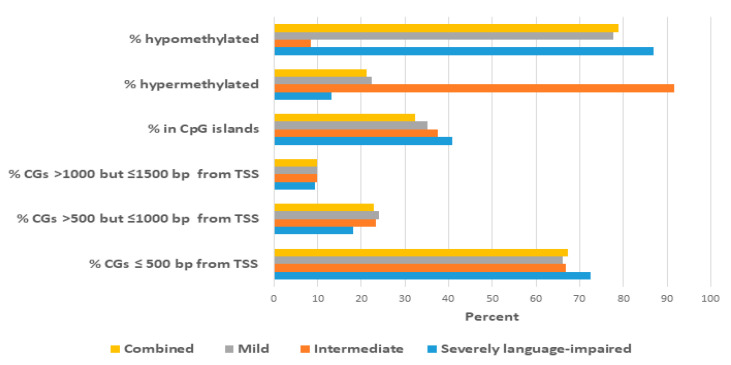
Summary of the proportions of differentially methylated CpG sites at different distances relative to the transcription start site (TSS) of the closest gene and the proportion of hypermethylated and hypomethylated sites in each ASD case group.

Figure 2B in comparison to that of the mild subgroup. It is notable that there are fewer significant DAGs in the combined case group compared to that of the mild subgroup (2570 vs. 4073), despite the larger number of individuals in the combined case group (292 vs. 149 case-control pairs). Figure 3 summarizes the location of the differentially methylated CpG sites relative to the transcription start site (TSS) for each case group and also the proportion of hypermethylated or hypomethylated sites in each group. In brief, more than 90% of CpG sites in all case groups were found within 1000 bp of the TSS, with the remainder less than 1500 bp away, suggesting that the majority of these sites are likely to be involved in the regulation of transcription. There are also noticeable quantitative differences in the methylation profiles among the case groups. For example, the severely language-impaired subgroup exhibits the greatest proportion of hypomethylated genes (86.8%) and the greatest proportion of CpGs (72.4%) that are closest (≤500 bp) to the TSS. By contrast, the intermediate subgroup exhibits the greatest proportion of hypermethylated genes (91.6%), while the location of the CpGs relative to the TSS is very similar to that of the mild and combined subgroups. Table S5 lists the map positions of the differentially methylated CpGs (and associated genes) in each subtype and the combined case group. 

**Figure 4:**
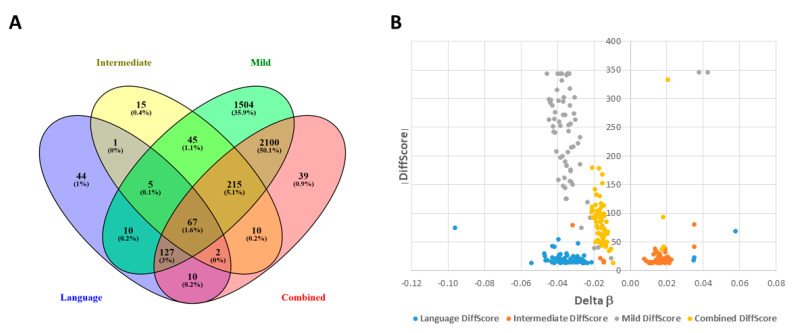
Stratification of ASD patients (*n* = 292) into distinct subphenotypes results in increased discovery of significant DAGs. (**A**) The Venn diagram shows unique significant DAGs that were identified using GenomeStudio Methylation Module v1.8 software with subphenotyping into three groups (mild, intermediate, and severely language-impaired) or without subphenotyping (combined case group) (FDR-adjusted *p*-values < 0.05). (**B**) Volcano plots for the 67 overlapping DAGs from each group, identified by color in the accompanying legend.

The Venn diagram in Figure 4A shows that there are 67 significant DAGs shared among the three subgroups and the combined case group, while Figure 4B shows volcano plots representing the relative distribution of these 67 DAGs in each group’s differential methylation profile. The differences in the distribution of these overlapping DAGs in each of the four groups reflect the differences that were revealed in Figure 2, with the majority of DAGs in the intermediate subgroup showing increased methylation, while these same DAGs in the severely language-impaired, mild, and combined groups show decreased methylation, as shown quantitatively in Figure 3. 

Not surprisingly, the |DiffScore| values (inversely related to *p*-values) for these DAGs are much greater in the mild subgroup than those for the severely language-impaired subgroup, which is likely the result of the larger number of samples in the mild subgroup (149 vs. 22 case-control pairs). On the other hand, despite having the largest number of cases and controls, the combined case group has smaller |DiffScore| and delta β values in comparison to the mild subgroup. This finding may reflect the increased heterogeneity underlying the combined case group in which the conglomeration of disparate cases dampens the average methylation differences (i.e., delta β) between the cases and controls. Hence, the present study demonstrates that phenotypic subtyping by clinical severity of ADI-R scores is a productive path for discovering a greater number of statistically significant DAGs between ASD cases and controls as well as differences in DNA methylation profiles among the subgroups.

### 2.2. Network Prediction Analyses of Subgroup-Associated DAGs

Ingenuity Pathway Analysis (IPA) was used to conduct functional analysis of the DAGs from each of the ASD subgroups as well as from the combined case group. Neurological functions enriched among DAGs in each subgroup and the combined case group are shown in Table 1. The specific DAGs associated with each function are included in Table S6. As shown, the severely language-impaired subgroup exhibits more functions known to be associated with ASD, such as: neuritogenesis, size and branching of neurites, and maturation of synapse and dendritic spines [21,22,23,24,25]. Figure S2 shows that axon guidance signaling and CXCR4 signaling are canonical pathways involved in the top network of genes involved in neuritogenesis. The intermediate subgroup is notably enriched in DAGs associated with the activation of neuroglia and astrocytes, suggesting inflammatory processes known to be involved in ASD [26,27]. Figure S3 shows that the neuroinflammation signaling pathway as well as the glucocorticoid signaling pathway are implicated by the genes involved in the abnormal morphology of neurons in the intermediate subgroup.

The mild subgroup is enriched in DAGs involved in sensory system development. Figure S4 shows that the top network of genes enriched for sensory system development is associated with axon guidance, transforming growth factor-β (TGFβ), and bone morphogenetic protein (BMP) signaling pathways. Interestingly, many individuals with ASD exhibit abnormal sensory responses, such as hypersensitivity to certain sounds, visual stimuli, taste, and textures [28,29,30]. Thus, it is not surprising that many genes related to the sensory system are affected. The nervous system functions associated with DAGs in the combined case group reflect those identified for the intermediate and mild subgroups but not for the severely language-impaired subgroup, which comprises just 7.5% of the total number of cases. 

With respect to neurological disorders (see Table 2, Table S7), DAGs in the severely language-impaired subgroup are enriched for genes contributing to comorbidities in ASD, such as cognitive impairment [31,32,33,34] and motor dysfunction [35,36]. While axon guidance and synaptogenesis signaling is implicated by the top network of genes associated with cognitive impairment (Figure S5), calcium signaling and dendritic cell maturation are indicated by the top network of genes involved in motor dysfunction (Figure S6). On the other hand, DAGs in the intermediate and mild subgroups as well as the combined case group are over-represented with respect to schizophrenia genes. Figure S7 shows that the neuroinflammation signaling pathway as well as the cAMP and G-protein coupled receptor signaling pathways are involved in schizophrenia in the intermediate subgroup, while synaptogenesis, GABA receptor, and CREB signaling in neurons are involved in the top network of genes in the mild subgroup (Figure S8). Genes associated with motor dysfunction and movement disorders are also over-represented among DAGs in the mild subgroup. Interestingly, only the severely language-impaired subgroup exhibits DAGs explicitly enriched for ASD or intellectual disability (ID), a comorbidity that presents more frequently in individuals with deficits in spoken language.

**Figure 5:**
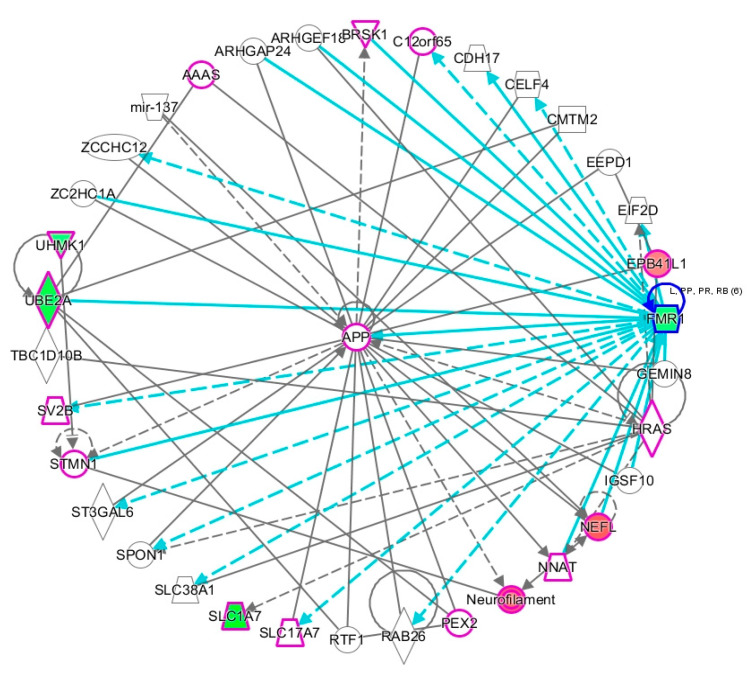
Gene network associated with DAGs enriched for ASD or intellectual disability. All genes involved in developmental disorder are outlined in purple. Genes colored red are hypermethylated while those colored green are hypomethylated. The turquoise colored lines indicate relationships between *FMR1* and other genes in the network. Solid lines denote direct interactions; dashed lines denote indirect interactions.

Figure 5 shows that one of the two networks of genes that are associated with ASD/ID includes *FMR1*, the gene responsible for fragile X syndrome, a genetic condition that is frequently associated with both intellectual disability and ASD. In a hierarchical layout of the network (Figure S9), *FMR1* is placed at the top of the network, highlighting its influence on the downstream genes, which include *UBE3A*, an E2 ubiquitin conjugating enzyme involved in cognitive disability, and *SLC1A7*, a glutamate transporter that is involved in pervasive developmental disorder (also used to describe ASD), social anxiety, and fragile X. 

### 2.3. Proximity of Hypermethylated and Hypomethylated CpGs to the TSS of the DAGs

Aside from identifying DAGs in each subtype, we also separately investigated the genes associated with hypermethylated and hypomethylated CpGs that were less than 500 bp from the TSS. Interestingly, two of the top genes associated with ASD or intellectual disability in the severely language-impaired subtype, *FMR1* and *UBE2A*, were among the hypomethylated genes closest to the TSS in this subgroup (Table 3). Other ASD-relevant genes within 500 bp of the TSS are *PAX8* and *SHANK1* (both hypomethylated), and *CADM1* and *PAX6* (both hypermethylated). PAX6 and PAX8 are members of the paired box (PAX) family of transcription factors. While PAX 6 is involved in modulating the fate of neural progenitor cells [37], genetic variants in *PAX8* are associated with sleep disturbance [38,39], a frequent comorbidity of ASD. SHANK1 is a scaffolding protein at the postsynaptic density that has been found to be involved in ASD [40]. Mutations in *CADM1*, which codes for a synaptic adhesion molecule, are also associated with ASD [41].

Table 4 shows the neurological functions and disorders enriched among the hyper- and hypomethylated genes with CpGs closest (<500 bp) to the TSS in the intermediate subgroup. Notable among this set of genes are the hypermethylated genes, *CORT* (corticostatin) and *NTF3* (neurotrophin 3), which are both involved in the loss of neurites. CORT is a neuropeptide that is involved in the depression of neuronal activity and the induction of slow-wave sleep, while NTF3 is a protein that controls the survival and differentiation of mammalian neurons. Among the ASD-relevant hypomethylated genes in this subgroup are *CTNNB1* and *GABRA3*. CTNNB1 (catenin beta1) plays a role in seizure susceptibility and cortical malformation as demonstrated in a *Ctnnb1* knock-out mouse model [42], and GABRA3, a GABA receptor that mediates fast inhibitory effects of GABA in the brain, is reduced in the cerebellum of individuals with ASD [43]. 

Among the top hypermethylated genes that are enriched in neurological functions and disorders in the mild subgroup are *ARX* and *CNTNAP2* (Table 5; Table 6). *ARX* (Aristaless related homeobox gene) is involved in a number of neurological diseases, including mental retardation, autism, epilepsy, and dystonia [44]. Its function as a homeobox gene is responsible for the wide range of neurological disease phenotypes that result from its mutation or dysregulation. *CNTNAP2*, a member of the neurexin family of proteins that serves as a cell adhesion molecule, is one of the most well-studied ASD risk genes [45,46,47,48,49]. Like *ARX*, mutations in *CNTNAP2* can lead to multiple neurological disease phenotypes, including autism, epilepsy, intellectual disability, obsessive compulsive disorder, and schizophrenia [50]. Notable among the hypomethylated genes in this subgroup are a number of chemokine genes, including *CCL1*, *CCL11*, *CCL2*, *CCL22*, *CCL5*, and *CCL7*. Not surprisingly, these DAGs are enriched among genes that are involved in the activation of neuroglia and neuroinflammation that have been associated with ASD [51,52]. Interestingly, hypomethylated genes associated with schizophrenia include a number of neurotransmitter receptors (e.g., cholinergic, cannabinoid, dopamine, GABA, glutamate, and serotonin) as well as ion channels and ion transporter proteins. These schizophrenia-associated DAGs in the mild ASD subgroup are significantly enriched for GABA receptor signaling (Fisher’s exact *p*-value = 1.98 × 10^−6^), neuroinflammation pathway signaling (*p* = 4.21 × 10^−5^, serotonin receptor signaling (1.75 × 10^−5^), calcium signaling (1.29 × 10^−3^), G-protein coupled receptor signaling (2.56 × 10^−3^), and glutamate receptor signaling (7.61 × 10^−3^) pathways. Overall, the proximity of the differentially methylated CpGs to the TSS of the DAGs enriched for neurological functions and diseases (as shown in Table 3, Table 4, Table 5 and Table 6) suggests that these sites may play a role in the transcriptional regulation of the associated genes.

### 2.4. Shared DAGs among Case Groups Converge on Inflammatory Responses

**Figure 6:**
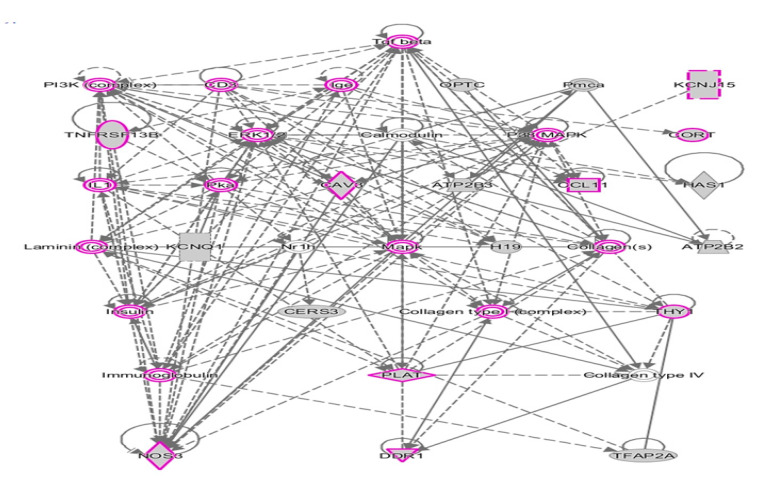
Hierarchical layout of top network of DAGs shared among 3 subphenotypes of ASD and the combined case group. Genes outlined in purple are involved in inflammatory responses.

We also used IPA to analyze the 67 DAGs (from Figure 4) shared by all three subgroups and the combined case group. Figure 6 shows the top network resulting from the network prediction analysis of the shared DAGs. This network is enriched in genes associated with inflammatory responses, suggesting that neuroinflammation may be a common theme underlying core features of ASD that are manifested in all subtypes. These results collectively demonstrate the value of reducing heterogeneity by subphenotyping individuals with ASD to maximize the ability to identify not only ASD-related DAGs but also ASD-associated functions, pathways, and disorders over-represented within each subgroup. Specifically, 1.62 times as many unique DAGs (4155) are identified among the three subphenotypes in comparison to that of the combined case group (2570). 

### 2.5. Overlap of DAGs and Differentially Expressed Genes (DEGs) from Analogous Phenotypic Subgroups from the Simplex Population

The subgroup-associated DAGs from the present methylation study were compared with DEGs from a separate study investigating transcriptomic data on individuals from the SSC cohort who were divided into subphenotypes using cluster analyses of ADI-R scores (Hu, V.W. and Bi, C., unpublished data). The overlapping genes for each subgroup and the combined case group included 12 DEGs from the severely language-impaired subgroup (hypergeometric cumulative *q*-value = 0.30), 8 DEGs from the intermediate subgroup (*q* = 0.35), 76 DEGs from the mild subgroup (*q* = 7.14 × 10^−4^, and 68 DEGs from the combined case group (*q* = 2.31 × 10^−4^) (Table 7). Thus, there is a significant overlap between DEGs and DAGs from the mild subgroup and combined case group but not from the severely language-impaired subgroup or intermediate subgroup, suggesting at least partial functional validation with regard to regulation of expression for the overlapping genes. It is not expected that all of the DEGs would be regulated by methylation differences between cases and controls. It should also be noted that there were fewer individuals in the transcriptomic investigation, with 7 case-control sibling pairs in the severely language-impaired subgroup, 26 pairs in the intermediate subgroup, 41 pairs in the mild subgroup, and 74 pairs in the combined case group. Furthermore, although the cases from the transcriptomic analysis were phenotypically representative of those from the three subgroups in this methylation study, the samples were not the same as those included in the present study.

### 2.6. Comparison with Other DNA Methylation Studies of ASD

DNA methylation has long been implicated as a contributor to the etiology of ASD-related disorders, such as Rett syndrome and Fragile X [53,54,55,56]. With respect to idiopathic ASD, we were the first to demonstrate that DNA methylation differences across multiple genes could be correlated to dysregulated expression of those genes in LCLs from discordantly diagnosed monozygotic twins and sibling pairs [7,57]. Since then, a number of other studies using blood-derived cells, buccal cells, and brain tissues have confirmed aberrant DNA methylation as a likely contributory factor to ASD [7,8,9,10,11,12,13,14,15]. However, there has been relatively low consistency with respect to the specific DAGs identified among the various studies, which is possibly due to the heterogeneity both within and among the cohorts used for the different analyses.

To our knowledge, this is the first study to undertake methylation analysis of ASD probands and unaffected siblings from the simplex population after dividing the cases into phenotypic subgroups to lessen the clinical heterogeneity inherent to ASD. A recent study that involved a meta-analysis of blood DNA methylation from two cohorts of individuals with ASD and controls did not find any DAGs that were significant after Bonferroni correction for multiple testing despite having 796 cases and 858 controls [58]. One of the cohorts included 343 probands and their respective sibling controls from the SSC. Among the 7 genes that were suggestively associated with ASD with *p*-value < 1 × 10^−5^, only one, *DIO3*, was found among DAGs from the mild subgroup tested here. Similarly, another recent methylation study using neonatal bloodspots from 1263 infants (of whom 50% were later diagnosed with ASD) concluded that ASD is not associated with robust differential methylation between the diagnosed children and the unaffected ones [59]. Another large methylome-wide association study (MWAS), which used cord blood from 701 8-year-olds and their respective scores on the Social and Communication Disorders Checklist as a measure of autistic traits, found no significant CpGs associated with the social traits [14]. Moreover, Massrali et al. [14] reported that a meta-analysis of the blood and blood spot data from the previous two MWAS studies [58,59] did not reveal any significant concordance in effect direction with their cord blood study; they therefore concluded that none of the MWAS studies identified any significant DAGs. It should be noted that all of these studies used methylation data collected on Illumina Infinium HumanMethylation450K BeadChip arrays, which offer greater potential for identifying differentially methylated CpG sites in comparison to the HumanMethylation27K array from which we derived the methylation data for our study. We therefore suggest that our ability to identify a large number of significant DAGs—some of which are replicated in different subgroups (or cohorts)—is due to the reduction in phenotypic or clinical heterogeneity among the cases in each subgroup. This interpretation is borne out by the smaller delta β values for DAGs when all the cases are combined into one group for methylation analysis. While we have used cluster analyses of ADI-R scores for phenotypic subgrouping in this study, heterogeneity reduction by genetic subgrouping (e.g., by *CHD8* mutations or by 16p11.2 deletions) has also resulted in enhanced ability to detect significant DAGs between ASD cases and controls [60]. In the same study, Siu et al. also reported no significant DAGs with a heterogeneous group of cases with idiopathic ASD, thereby reaffirming the value of heterogeneity reduction in genome-wide DNA methylation studies of ASD. Aside from the subgrouping methods discussed above, heterogeneity reduction in ASD can also be accomplished by subtyping individuals by associated comorbidities, such as intellectual disability, immune dysfunction, or gastrointestinal disease.

### 2.7. Advantages and Limitations of Study Design and Future Considerations

This study examines the impact of applying clinical subtyping to methylation analyses of males with ASD from the simplex population. While the inclusion of only males eliminates sex as a confounding factor, future studies should also include females to investigate the potential for sex-related differences in DNA methylation. The main limitation here is the relatively small number of cases studied, particularly in the severely language-impaired subgroup, which represents the smallest phenotypic subgroup identified by ADI-R cluster analyses of cases from simplex families. Despite this limitation, the severely language-impaired subgroup exhibited the largest differences in β values (and fold-change) between cases and controls relative to that of the other groups, perhaps reflecting the heightened clinical severity of this subgroup. Furthermore, network prediction analyses show that this subgroup was most enriched in neurological functions and comorbidities associated with ASD and was also the sole subgroup enriched for genes directly involved in autism and intellectual disability. 

Another limitation is that we were not able to confirm the DAGs identified in each subgroup inasmuch as this bioinformatics study focused on a re-analysis of existing methylation data, and we did not have access to the samples for pyrosequencing analyses. Future studies should therefore address the confirmation of these results not only with regard to methylation analyses of specific DAGs, but also with respect to application of this subtyping method to methylation analyses of an additional cohort of individuals with ASD from the simplex population, preferably with more CpG sites interrogated. Nevertheless, the overlap of DAGs between and among the three phenotypic subgroups represents replication of at least these specific DAGs in different cohorts, as there is no overlap of individuals among the subgroups. In addition, the overlap of DAGs from this study and DEGs from a separate transcriptomic study involving analogous subgroups from the simplex population offers functional support for a fraction of the DAGs identified here. Finally, the Infinium HumanMethylation27K BeadChip array, which was used to generate the methylation data analyzed in this study, is also a limitation in terms of the relatively low number of CpGs interrogated. More recent BeadChip arrays currently probe over 800K CpG sites, and whole genome bisulfite sequencing can assess even more. Thus, in light of our study demonstrating increased discovery of significant DAGs as well as ASD-associated neurological functions and disorders as a result of phenotypic subgrouping, it will be of interest for future studies to analyze more comprehensive methylation data in the context of ASD subtypes to confirm the main findings reported here. 

## 3. Materials and Methods 

### 3.1. Acquisition of Methylation Data for Individuals with ASD from the Simplex Population

DNA methylation data from a study of individuals with ASD and their unaffected siblings who were included in the Simons Simplex Collection (SSC) (New York, NY, USA) were downloaded from the National Database for Autism Research (NDAR). NDAR is a repository of clinical, omics, and brain imaging data from autism studies that is maintained by the NIMH Data Archives (NDA) (Rockville, MD, USA). The original data were deposited by Dr. Stephen Warren (Emory University, Atlanta, GA, USA) for a methylation pilot study entitled “Epigenetic marks as peripheral biomarkers for autism” (Study ID: #287). For the pilot study, DNA methylation for over 300 simplex cases and their respective sibling controls was analyzed on Illumina Infinium HumanMethylation27K BeadChips covering 27,578 CpG dinucleotides, with the raw data deposited into NDAR.

### 3.2. Phenotypic Subtyping for ASD Individuals from Simplex Families 

The Autism Diagnostic Interview-Revised (ADI-R), which is considered a gold-standard diagnostic tool for autism, is based on a series of questions posed to parents or primary caregivers that interrogate a subject’s performance on a wide range of behaviors impacted by ASD [61]. These behaviors are scored for severity by a trained neuropsychologist according to the parent/caregiver’s response. Raw ADI-R scoresheets for 1900 individuals with ASD (i.e., probands) were obtained from the SSC. As described previously for multiplex families [16], 123 severity scores on 63 ADI-R items (see Table S8) for each individual were subjected to K-means cluster (KMC) analyses, which showed that K = 3 (representing separation into 3 subgroups) resulted in an optimum separation of cases with distinguishable severity profiles. Based on these profiles, the three subgroups were described as mild, intermediate, and severely language impaired, which are similar to three of the four subgroups previously identified in multiplex families [16]. The fourth subgroup in the multiplex population, which exhibited a noticeably higher frequency of savant skills, was not clearly discernible within the simplex population. 

The sample identification numbers (IDs) of the probands from the Warren pilot methylation study were then cross-referenced against the sample IDs associated with 1900 ADI-R scoresheets of individuals with ASD from the SSC. Based on the severity profiles that resulted from the ADI-R cluster analyses, 292 cases (all males) with available methylation data were stratified into three subphenotypes as follows: mild (*n* = 149), intermediate (*n* = 121), and severely language impaired (*n* = 22). Differences in the severity profiles of the 292 individuals selected for our study were verified by hierarchical clustering (HCL) and principal components analyses (PCA) using open-access Multiexperiment Viewer software [62]. The demographic information on individuals included in the current study is presented in Table S9. 

### 3.3. Identification of Differentially Methylated Regions (DMR) and DMR-Associated Genes (DAG)

Raw signal intensities were extracted from idat files derived from the Illumina Infinium HumanMethylation27K BeadChip analyses using Illumina’s GenomeStudio Methylation Module v1.8 (Illumina, San Diego, CA, USA). The DNA methylation level of each interrogated CpG site is reported in the GenomeStudio software as an average β value, ranging from 0 (completely unmethylated) to 1 (100% methylated). The β value is defined as the ratio of signal from the methylated probe (M) to the sum of the signals from the methylated probe (M) and unmethylated probe (U) plus 100, or β = M/(M + U + 100). Analysis of differential methylation between ASD cases and sibling controls was performed in GenomeStudio using the Illumina Custom Model. This error model developed by Illumina assumes a normal distribution of the β values among biological replicates and results in a differential methylation score (DiffScore) for each interrogated CpG site. Using the absolute value of DiffScores reported by the GenomeStudio software, *p*-values were calculated with the following formula: *p* = 10^−(|DiffScore|/10)^. |DiffScore| > 13.0103 is equivalent to a *p*-value < 0.05. Correction for multiple comparisons was accomplished by computing the false discovery rate (FDR), which is integrated into the GenomeStudio software. Interrogated CpG sites were annotated in GenomeStudio with respect to their corresponding genes. DAGs with FDR-adjusted *p* < 0.05 were classified as significant. The three ASD subgroups were analyzed separately as well as in combination (i.e., combined case group) for DNA methylation differences when compared to their respective sibling controls. Volcano plots showing the overall distribution of significant DAGs for each subgroup or the combined case group were generated by plotting |DiffScore| against delta β, i.e., (β_case_ – β_control_). The distance of the differentially methylated CpGs to the TSS of the nearest gene was obtained from Illumina’s content file for the HumanMethylation27K BeadChip array, which provides the mapping information for each CpG as well as its location with respect to CpG islands. All of the CpG sites were within 1500 bp of a TSS, suggesting their potential involvement in the regulation of gene expression.

### 3.4. Network Prediction Analyses of DAGs

Ingenuity Pathway Analysis (IPA) network prediction software (Qiagen, Germantown, MD, USA) was used to identify enriched functions, pathways, and disorders associated with DAGs from the methylation analyses based on Fisher exact *p*-values of *p* ≤ 0.05, using genes in IPA’s Knowledgebase as the reference set of genes to determine enrichment in pathways or functions among the DAGs.

### 3.5. Hypergeometric Distribution Analyses 

Hypergeometric distribution analyses were employed to identify the significance of overlap between DAGs and differentially expressed genes (DEGs) from analogous phenotypic subgroups of ASD from a separate study (Hu, V.W. and Bi, C., unpublished data), which involved re-analysis of transcriptomic data from a previously published study that used LCLs from the SSC [63]. The overlapping genes were identified using an open-access Venn diagram software program called Venny 2.1.0 (https://bioinfogp.cng.csic.es/tools/venny/) [64]. Significant overlap between the DAGs and DEGs was determined by hypergeometric distribution analyses using the open-access CASIO Keisan Online Calculator (http://keisan.casio.com/exec/system/1180573201), with significance determined by an upper cumulative *q*-value of ≤ 0.05. Venny 2.1.0 was also used to identify overlap among subgroup-associated DAGs and those from the combined case group.

## 4. Conclusions

This is the first study to investigate differential methylation in individuals with ASD from the simplex population who were divided into distinct phenotypic subgroups by cluster analyses of ADI-R scores. This study is important because it demonstrates a link between DNA methylation and the etiology of ASD in this population. We suggest that subphenotyping enables more efficient identification of statistically significant DAGs which, in turn, reveal subphenotype-dependent functions and comorbidities that are associated with each ASD subgroup. Such discrimination of the biological differences between ASD subphenotypes is essential to our understanding of the complex pathobiology of ASD as well as our ability to develop targeted ASD subtype-directed therapeutics.

## Figures and Tables

**Table 1:** Significantly over-represented neurological functions among DAGs from three phenotypic subgroups of ASD and a combined case group.

Nervous System Development and Function Severely Language Impaired (*n* = 22)	*p*-Value *	Number of Genes
Abnormal morphology of neurons	2.21 × 10^−4^	19
Sensorimotor integration	3.39 × 10^−3^	2
Neuritogenesis	3.52 × 10^−3^	19
Maturation of synapse	4.25 × 10^−3^	3
Size of neurites	6.09 × 10^−3^	2
Branching of neurites	6.31 × 10^−3^	11
Maturation of dendritic spines	7.15 × 10^−3^	2
Development of neurons	9.24 × 10^−3^	22
**Intermediate (*n* = 121)**		
Activation of neuroglia	7.35 × 10^−5^	11
Activation of astrocytes	4.77 × 10^−4^	6
Abnormal morphology of neurons	1.22 × 10^−3^	21
Abnormal morphology of axons	1.27 × 10^−3^	7
Abnormal morphology of neurites	1.48 × 10^−3^	9
Loss of neurites	2.91 × 10^−3^	4
**Mild (*n* = 149)**		
Abnormal morphology of nervous system	4.34 × 10^−15^	297
Sensory system development	7.77 × 10^−15^	194
**Combined (*n* = 292)**		
Abnormal morphology of nervous system	4.37 × 10^−11^	194
Sensory system development	6.17 × 10^−9^	121
Abnormal morphology of neurons	2.26 × 10^−8^	121
Activation of neuroglia	4.27 × 10^−8^	45

* Fisher exact *p*-value representing the probability that the indicated function is not over-represented among the DAGs for each group, using all genes in IPA’s Knowledgebase as the reference gene set.

**Table 2:** Significantly over-represented neurological and developmental disorders among DAGs from the three phenotypic subgroups of ASD and the combined case group.

**Neurological Diseases**	*p*-Value *	**Number of Genes**
**Severely language impaired**		
Cognitive impairment	1.32 × 10^−5^	26
Syndromic X⁻linked mental retardation	2.99 × 10^−4^	6
Mental retardation	4.13 × 10^−3^	14
Motor dysfunction or movement disorder	7.75 × 10^−3^	29
Autosomal dominant mental retardation type 11	9.94 × 10^−3^	1
**Intermediate**		
Schizophrenia	7.73 × 10^−4^	20
Demyelination of nerves	4.59 × 10^−3^	4
**Mild**		
Schizophrenia	1.18 × 10^−16^	184
Motor dysfunction or movement disorder	5.98 × 10^−14^	393
Movement Disorders	3.93 × 10^−13^	384
**Combined**		
Schizophrenia	1.81 × 10^−7^	106
**Developmental Disorders (Severely Language Impaired Only)**	*p*-Value *	**Number of Genes**
Autism or intellectual disability	3.31 × 10^−3^	15
Autism spectrum disorder or intellectual disability	3.67 × 10^−3^	17
Dystrophy of muscle	5.51 × 10^−3^	10

* Fisher exact *p*-value representing the probability that the indicated disorder is not over-represented among the DAGs for each group, using all genes in IPA’s Knowledgebase as the reference gene set.

**Table 3:** Neurological functions and diseases enriched among DAGs implicated by CpGs within 500 bp of the TSS in the severely language-impaired subgroup.

**Nervous System Development & Function**	*p*-Value *	**DAGs (<500 bp from TSS)**
***Severely language-impaired subtype***		***Hypermethylated***
Cell-cell adhesion of neurons	2.29 × 10^−4^	CADM1, NINJ2
Induction of neural crest	2.37 × 10^−3^	TFAP2A
Scaffolding of postsynaptic region	3.55 × 10^−3^	CADM1
Corticogenesis	3.55 × 10^−3^	PAX6
Quantity of neurons	7.25 × 10^−3^	CADM1, NPTX2, PAX6, TFAP2A
Abnormal morphology of brain	7.37 × 10^−3^	CRMP1, EPB41L1, PAX6, TFAP2A
Size of growth cone	1.65 × 10^−2^	PAX6
Morphology of nervous system	3.71 × 10^−2^	CADM1, CRMP1, EPB41L1, PAX6, TFAP2A
Memory consolidation	3.73 × 10^−2^	PAX6
Quantity of nerve ending	3.73 × 10^−2^	CADM1
Development of sensory neurons	3.95 × 10^−2^	PAX6
Abnormal morphology of forebrain	4.58 × 10^−2^	PAX6, TFAP2A
***Severely language-impaired subtype***		***Hypomethylated***
Abnormal morphology of sensory neurons	6.21 × 10^−4^	ATP2B2, CETN2, KCNQ1, OPN4, PAX8
Delay in initiation of maturation of interneurons	5.93 × 10^−3^	FMR1
Abnormal morphology of neurons	6.49 × 10^−3^	ATP2B2, CABP4, CCL11, CETN2, CORT, FMR1, KCNQ1, NOS3, OPN4, PAX8, SHANK1
Long term synaptic depression of hippocampal cells	9.57 × 10^−3^	FMR1, INS
Circadian phase shifting	1.18 × 10^−2^	OPN4
Remodeling of dendrites	1.18 × 10^−2^	FMR1
Density of GABAergic synapse	1.18 × 10^−2^	FMR1
**Neurological Diseases**	*p*-Value *	**DAGs (<500 bp from TSS)**
***Severely language-impaired subtype***		***Hypermethylated***
Autosomal dominant mental retardation type 11	1.19 × 10^−3^	EPB41L1
Cognitive impairment	1.33 × 10^−3^	CRMP1, EPB41L1, GSTM1, NPTX2, PAX6, TFAP2A
Epileptic seizure	3.70 × 10^−2^	CCN1, NPTX2
Mental retardation	4.09 × 10^−2^	EPB41L1, PAX6, TFAP2A
Abnormal morphology of forebrain	4.58 × 10^−2^	PAX6, TFAP2A
***Severely language impaired subtype***		***Hypomethylated***
Syndromic X-linked mental retardation	2.20 × 10^−5^	BCAP31, FMR1, PHF8, SLC1A7, SLC9A6, UBE2A
Cognitive impairment	1.65 × 10^−3^	BCAP31, FMR1, GRM4, INS, KCNQ1, LRRN4, NOS3, PDZK1, PHF8, POLR3C, SHANK1, SLC1A7, SLC9A6, UBE2A, UHMK1
Fragile X-associated tremor ataxia syndrome	5.93 × 10^−3^	FMR1
Fragile X syndrome with Prader-Willi-like phenotype	5.93 × 10^−3^	FMR1
Movement Disorders	1.15 × 10^−2^	ATP2B2, ATP2B3, BCAP31, C9, CCL11, CETN2, COL6A3, F8A1, FCGR3A/FCGR3B, FMR1, KCNQ1, MYH7, OPN4, SDC4, SGCG, SLC17A4, SLC1A7, SLC9A6, TGM6

* Fisher exact *p*-value indicating the probability that the function or disorder is not enriched among the DAGs for this subgroup.

**Table 4:** Neurological functions and diseases enriched among DAGs implicated by CpGs within 500 bp of the TSS in the intermediate subgroup.

**Nervous System Development & Function**	*p*-Value *	**DAGs (<500 bp from TSS)**
***Intermediate subtype***		***Hypermethylated***
Loss of neurites	4.64 × 10^−4^	CORT, GJB1, NTF3, SERPINA3
Activation of neuroglia	1.11 × 10^−3^	C1QA, CCL11, CCL22, FGF1, GJB1, NOS3, SMPD3
Abnormal morphology of neurons	1.22 × 10^−3^	ATP2B2, C1QA, CABP4, CCL11, CORT, GJB1, KCNQ1, NOS3, NTF3, OPN4, PAX8, PLP1, RHO, SERPINA3, SORBS2
Abnormal morphology of nerve ending	1.66 × 10^−3^	C1QA, NTF3
Abnormal morphology of sensory neurons	2.16 × 10^−3^	ATP2B2, KCNQ1, NTF3, OPN4, PAX8
Loss of axons	2.38 × 10^−3^	CORT, GJB1, NTF3
Activation of astrocytes	3.04 × 10^−3^	C1QA, CCL11, FGF1, SMPD3
Formation of excitatory synapses	3.22 × 10^−3^	NTF3, SORBS2
Abnormal morphology of neurites	5.98 × 10^−3^	CORT, GJB1, NTF3, PLP1, SERPINA3, SORBS2
Evoked potential	6.24 × 10^−3^	ATP2B2, KCNQ1, NTF3, PAX8
Abnormal morphology of nervous system	6.49 × 10^−3^	ATP2B2, C1QA, CABP4, CCL11, CNGA2, CORT, FGF1, GJB1, KCNQ1, NOS3, NR5A1, NTF3, OPN4, PAX8, PLP1, RHO, SERPINA3, SMPD3, SORBS2
***Intermediate subtype***		***Hypomethylated***
Morphology of brain	8.83 × 10^−4^	ARSA, CTNNB1, GBX2, GSX1, TFAP2A
Differentiation of sensory progenitor cells	9.40 × 10^−4^	CTNNB1
Neurogenesis of dopaminergic neurons	1.88 × 10^−3^	CTNNB1
Abnormal morphology of forebrain	2.42 × 10^−3^	ARSA, GSX1, TFAP2A
Abnormal morphology of brain	3.12 × 10^−3^	ARSA, GBX2, GSX1, TFAP2A
Auditory evoked potential	3.15 × 10^−3^	ARSA, KCNQ1
Cell survival of dopaminergic neurons	3.76 × 10^−3^	CTNNB1
Formation of forebrain	3.77 × 10^−3^	CTNNB1, GBX2, GSX1
Accumulation of microglia	7.50 × 10^−3^	CTNNB1
Lack of cerebellum	8.43 × 10^−3^	GBX2
**Neurological Diseases**	*p*-Value *	**DAGs (<500 bp from TSS)**
***Intermediate subtype***		***Hypermethylated***
Abnormal morphology of mechanosensory neurons	8.56 × 10^−4^	ATP2B2, KCNQ1, NTF3, PAX8
Lack of muscle sensory neurons	7.85 × 10^−3^	NTF3
***Intermediate subtype***		***Hypomethylated***
Early-onset neurological disorder	3.20 × 10^−4^	ARSA, GABRA3, KCNQ1, PDZRN3
Cognitive impairment	3.54 × 10^−4^	ARSA, CTNNB1, GABRA3, GSTM1, KCNQ1, TFAP2A
Autosomal dominant mental retardation type 19	9.40 × 10^−4^	CTNNB1
Early-onset schizophrenia	1.13 × 10^−3^	GABRA3, PDZRN3
Lack of cerebellum	8.43 × 10^−3^	GBX2

* Fisher exact *p*-value indicating the probability that the function or disorder is not enriched among the DAGs for this subgroup.

**Table 5:** Neurological functions enriched among DAGs implicated by CpGs within 500 bp from the TSS in the mild subgroup.

Nervous System Development & Function	*p*-Value *	DAGs (<500 bp from TSS)
***Mild subtype***		***Hypermethylated***
Development of striatum	1.26 × 10^−6^	ARX, CNTNAP2, GSX1, PAX6
Abnormal morphology of nervous system	3.61 × 10^−6^	ARX, ATP8A2, CDH11, CNTNAP2, EDN3, FOXB1, FST, GSX1, OSTM1, PAX6, RBP1, SYN2, TFAP2A, TLX3, ZIC1
Abnormal morphology of glutamatergic neuron	4.96 × 10^−5^	PAX6,TLX3
Migration of neurons	5.84 × 10^−5^	ARX, CNTNAP2, ERRFI1, FLRT2, LRP12, PAX6, TLX3
Development of neurons	9.20 × 10^−5^	ARX, ATP8A2, CADM1, CCN1, CDH1, CNTNAP2, FLRT2, ITGA1, LRP12, PAX6, SYN2, TLX3, TPBG
Quantity of neurons	1.19 × 10^−4^	ARX, CADM1, CNTNAP2, ENTPD3, FST, PAX6, PTGER2, SYN2, TFAP2A
Abnormal morphology of brain	1.23 × 10^−4^	ARX, CNTNAP2, FOXB1, GSX1, OSTM1, PAX6, SYN2, TFAP2A, ZIC1
Abnormal morphology of forebrain	1.94 × 10^−4^	ARX, CNTNAP2, FOXB1, GSX1, PAX6, TFAP2A
Growth of cerebellum	2.30 × 10^−4^	FOXB1, ZIC1
Cell cycle progression of neurons	9.71 × 10^−4^	PAX6, ZIC1
Growth of brain	1.22 × 10^−3^	ARX, FOXB1, PAX6, ZIC1
Developmental process of synapse	1.83 × 10^−3^	CADM1, CDH1,FLRT2, SYN2, TPBG
Initiation of migration of neurons	2.90 × 10^−3^	ARX
Formation of forebrain	3.02 × 10^−3^	ARX, CNTNAP2, GSX1, PAX6, ZIC1
Synaptic transmission of hippocampal CA1 region	4.14 × 10^−3^	CNTNAP2, SYN2
Development of sensory neurons	4.39 × 10^−3^	PAX6, TLX3
Association of synaptic vesicles	5.80 × 10^−3^	SYN2
***Mild subtype***		***Hypomethylated***
Sensory system development	6.57 × 10^−9^	ABCA4, ADCY1, AIPL1, AIRE, ALDH1A2, ALOX15, APOB, AQP1, ARSG, ASPA, ATP2B2, BBS1, BBS7, BCL9L, BFSP2, BMPR1B, BRD1, C5AR1, CABP4, CCL1, CDH5, CDK20, CNGA3, COL18A1, COL1A1, COL8A2, CPLX4, CRX, CRYAA/CRYAA2, CRYAB, CRYBA4, CRYBB2, CRYGA, CRYGB, CRYGC, CXCR3, DNMT3A, DPT, DSC1, EGFR, ELOVL4, EMX1, FABP7, FASLG, FEZF2, FGF1, FGF7, FGFR2, FYCO1, GDF3, GFRA1, GJA3, GNAT2, GRK1, GUCA1A, GUCY2F, HCN1, HGF, HK2, HRG, IL1R1, IRX3, KERA, KNG1, KRT12, KRT4, LCK, LCTL, LHX2, LRAT, LRP8, LYVE1, MARCKSL1, METRN, MFAP2, MFRP, MGAT5, MSX2, NEUROD2, NEUROD4, NOTCH3, NRTN, NTF3, NTF4, NXNL1, OLIG2, OPN4, PDCD1, PDE6B, PDE6C, PF4V1, PLA2G3, POR, POU4F2, POU4F3, PPT2, PRPH2, PTGS2, PTPRS, PYGO1, RB1, RBP3, RDH8, RHO, RPE65, RUNX3, S1PR3, SERPINF1, SIX3, SIX6, SLC17A8, SLC39A5, SOX10, SOX11, SPI1, TFB1M, TH, THBS1, TP63, TRPV4, TUB, TULP1, TYRP1, USH2A, VCAM1, VSX2, WNT2
Activation of neuroglia	1.35 × 10^−8^	ABCA4, ADIPOQ, AGT, ALB, C1QA, C5AR1, CCL1, CCL11, CCL21, CCL22, CCL5, CCL7, CD40LG, CHGA, CNGA3, CSF3, DRD2, EGFR, F2, FASLG, FGA, FGF1, FGG, GFAP, GJB1, GJC2, GRK2, IL10, IL1R1, MC4R, MOG, MSTN, MYOD1, NOS3, NR4A1, NRG1, PDE6B, PDK4, PTGS2, RDH8, SERPINF2, SLC6A4, SST, TLR7, TLR9, TREM2, TRPM2, VTN

* Fisher exact *p*-value indicating the probability that the function is not enriched among the DAGs for this subgroup.

**Table 6:** Neurological diseases enriched among DAGs implicated by CpGs within 500 bp from the TSS in the mild subgroup.

Neurological Diseases	*p*-Value *	DAGs (<500 bp from TSS)
***Mild subtype***		***Hypermethylated***
Cerebellar ataxia with intellectual disability	2.95 × 10^−4^	ATP8A2, PAX6
Movement Disorders	6.67 × 10^−4^	ARX, ATP8A2, CDH11, DKK3, ERRFI1, FGF12, FLRT2, GABRE, GYPC, NKX6-2, PAX6, PDE4B, SYN2, ZIC1
Epilepsy	7.73 × 10^−4^	ARX, CCN1, CNTNAP2, ERRFI1, FGF12, GABRE, SYN2
Epilepsy or neurodevelopmental disorder	1.08 × 10^−3^	ARX, CCN1, CNTNAP2, EDN3, ERRFI1, FGF12, GABRE, SYN2
ARX-related X-linked mental retardation	2.90 × 10^−3^	ARX
Moderate to severe stage mental retardation	2.90 × 10^−3^	ARX
Severe hypotonia	2.90 × 10^−3^	ARX
Susceptibility to autism type 15	2.90 × 10^−3^	CNTNAP2
Cerebellar ataxia, mental retardation, and dysequilibrium syndrome type 4	2.90 × 10^−3^	ATP8A2
Autism	3.95 × 10^−3^	ARX, CNTNAP2, GABRE
Seizures	4.52 × 10^−3^	ARX, CCN1, CNTNAP2, ERRFI1, FGF12, GABRE, SYN2
Familial pervasive developmental disorder	4.64 × 10^−3^	ARX, CNTNAP2
Ataxia	5.23 × 10^−3^	ARX, ATP8A2, NKX6-2, PAX6, ZIC1
***Mild subtype***		***Hypomethylated***
**Schizophrenia**	9.17 × 10^−11^	ACSBG1, ADRA2B, APOA4, APOB, ATP1A4, ATP2B2, ATP4A, ATP4B, BPIFC, CA1, CA5A, CA7, CA9, CAD, CALY, CAP2, CCDC60, CCK, CCKAR, CHI3L1, CHRM1, CHRNA1, CHRNA2, CHRNA9, CHRNB4, CNR1, COL3A1, CPLX2, CRHBP, CYP2D6, CYP2E1, CYP3A5, DAB1, DAO, DDR1, DLG2, DRD1, DRD2, DRD5, DRP2, EGFR, ERBB4, FABP7, FAM3D, FCGR2A, FCGR3A/FCGR3B, GABRA3, GABRA5, GABRA6, GABRG3, GABRP, GABRR1, GFAP, GPR37, GRIK1, GRIK5, GRIN1, GRIN3A, GRM4, GRM7, HIPK3, HRH1, HTR2B, HTR3A, HTR3B, HTR3C, HTR3D, HTR3E, HTR5A, HTR6, INS, LAMA1, LGALS1, MAGEC1, MC4R, MEST, MT2A, MTNR1B, NEFM, NELL1, NOTCH4, NPAS3, NRG1, NRXN1, NTF3, NTNG2, OFCC1, OXTR, PDZRN3, PLP1, PMP22, POMC, PRL, PTGS2, RCAN2, RIT2, RPP21, SCG2, SCG5, SCN2B, SCN3B, SCN4A, SCN9A, SLC14A1, SLC18A1, SLC18A2, SLC31A2, SLC5A7, SLC6A4, SLC7A11, SOX10, SST, STON1, SYN3, SYT3, SYT4, TAC1, TF, THBS1, TRAK1, TTR, UGT1A3, XDH

* Fisher exact *p*-value indicating the probability that the neurological disease is not enriched among the DAGs for this subgroup.

**Table 7:** Overlapping genes among DAGs and Differentially Expressed Genes (DEGs) from the three phenotypic subgroups of ASD and the combined case group.

Overlap between DAGs and DEGs in Language-Impaired Subgroup (hypergeom. *q*=0.30)	Overlap between DAGs and DEGs in Intermediate Subgroup (*q* = 0.35)	Overlap between DAGs and DEGs in Mild Subgroup (*q* = 7.14 × 10^−4^)		Overlap between DAGs and DEGs in Combined Case Group (*q* = 2.31 × 10^−4^)	
ALOX15B	AIM2	AADAC	LIMS2	AIM2	LCN2
CARD14	ARSA	AMPH	MATK	ASB10	LHX3
CAV3	ATP8B1	APOB	MEP1B	ASCL2	LYST
CCL11	KCNQ1	BNC1	MMP10	BGN	MAGEA11
COL6A3	LDHC	C15orf32	MS4A12	BMP10	MAGEA8
CTRB1	NEFL	C19orf18	MS4A6A	BMX	MEPE
FAM83A	RGS3	C8B	NKX6-2	BTNL2	MS4A12
HIPK4	SMPD3	CADPS	NNAT	C14orf39	MS4A6A
HSPB8		CCDC54	NR1I3	C15orf32	NR1I3
PSG4		CDH5	NR4A1	CARD14	NR5A1
SEMA3B		CEACAM3	NRXN1	CCR9	OR10A4
WFDC8		CHI3L1	NRXN3	CHRNA1	OR10H1
		COX4I2	OR10H1	CNKSR1	OR1N1
		CPA6	P4HA3	CYP2C9	OR3A3
		DCN	PCDHB7	CYP4F3	P4HA3
		DLK1	PHYHIP	DEFB126	PABPC5
		DNMT3L	RGS12	EGFL7	PLAT
		DOCK1	S100A3	FBLN1	PMCHL1
		EDNRB	SATB1	FLRT2	PNLIPRP1
		FMR1NB	SCARF1	GALR1	RASL12
		FRMPD2	SCN3B	GATA5	RGS13
		FXYD3	SLC13A5	GKN1	RHOJ
		GATA5	SLC35E4	GLIS1	SCGB1D1
		GIPR	SLC6A11	GRIK2	SLC6A11
		GLIS1	SPATA3	GUCA2B	SPATA21
		GUCA2B	SPINK4	GUCY2F	SPRR3
		HBE1	SPOCK3	HCRTR1	SPTB
		IFNA16	SPTB	IFNA17	TFAP2B
		IQCF1	ST6GAL2	IGSF11	TRPM5
		KLHDC7A	SYN3	IQCF1	TTLL2
		KLK3	SYNE2	KIF25	WFDC5
		KLK9	TMEM119	KLK9	WWP1
		KRTAP20-1	TMEM40	KRT1	ZBED2
		LAD1	TNNI1	LCE3C	ZDHHC11
		LCE3C	TRIM16		
		LCN2	USH2A		
		LHX3	ZDHHC11		
		LILRA5	ZPLD1		

## Supplementary Materials

The following are available online at https://www.mdpi.com/1422-0067/21/18/6877/s1, Figure S1. |DiffScore| vs. log2 Fold-change values for all groups; Figure S2. Gene network associated with DAGs involved in neuritogenesis in the severely language-impaired subgroup; Figure S3. Gene network associated with DAGs involved in abnormal morphology of neurons in the intermediate subgroup; Figure S4. Gene network associated with DAGs involved in sensory system development in the mild subgroup; Figure S5. Gene network associated with DAGs involved in cognitive impairment in the severely language-impaired subgroup; Figure S6. Gene network associated with DAGs involved in motor dysfunction in the severely language-impaired subgroup; Figure S7. Gene network associated with DAGs involved in schizophrenia in the intermediate subgroup; Figure S8. Gene network associated with DAGs involved in schizophrenia in the mild subgroup; Figure S9. Hierarchical layout of a top gene network of DAGs associated with ASD and ID in the severely language-impaired subgroup; Table S1. Significant DAGs associated with the severely language-impaired subgroup; Table S2. Significant DAGs associated with the intermediate subgroup; Table S3. Significant DAGs associated with the mild subgroup; Table S4. Significant DAGs associated with the combined case group; Table S5. Map positions (distance from TSS) of the differentially methylated CpG sites in each subtype and the combined case group; Table S6. Over-represented neurological functions among DAGs in all subgroups and the combined case group; Table S7. Over-represented neurological and developmental disorders among DAGs in all subgroups and the combined case group; Table S8. List of ADI-R items with score adjustments used for cluster analyses, as described in [16]; Table S9. Demographic information for probands and siblings used in this study.
