# Supplementary material for: Phenotypic Subtyping and Re-Analysis of Existing Methylation Data from Autistic Probands in Simplex Families Reveal ASD Subtype-Associated Differentially Methylated Genes and Biological Functions
Source: Int J Mol Sci. 2020 Sep 19;21(18):6877. doi: 10.3390/ijms21186877 (PMC7555936; doi:10.3390/ijms21186877)
Supplement: Supplementary file 1 [file ijms-21-06877-s001.zip › ijms-927106-supplementary/Suppl. Materials_revised MS/Supplementary Figures S1-S9.pdf]

List of Supplementary Figures

Figure S1. |DiffScore| vs. log2 Fold-change values for all groups

Figure S2. Gene network associated with DMGs involved in neuritogenesis in the severely language-impaired subgroup

Figure S3. Gene network associated with DMGs involved in abnormal morphology of neurons in the intermediate subgroup

Figure S4. Gene network associated with DMGs involved in sensory system development in the mild subgroup

Figure S5. Gene network associated with DMGs involved in cognitive impairment in the severely language-impaired subgroup

Figure S6. Gene network associated with DMGs involved in motor dysfunction in the severely language-impaired subgroup

Figure S7. Gene network associated with DMGs involved in schizophrenia in the intermediate subgroup

Figure S8. Gene network associated with DMGs involved in schizophrenia in the mild subgroup

Figure S9. Hierarchical layout of a top gene network of DMGs associated with ASD and ID in the severely language-impaired subgroup

Fig. S1

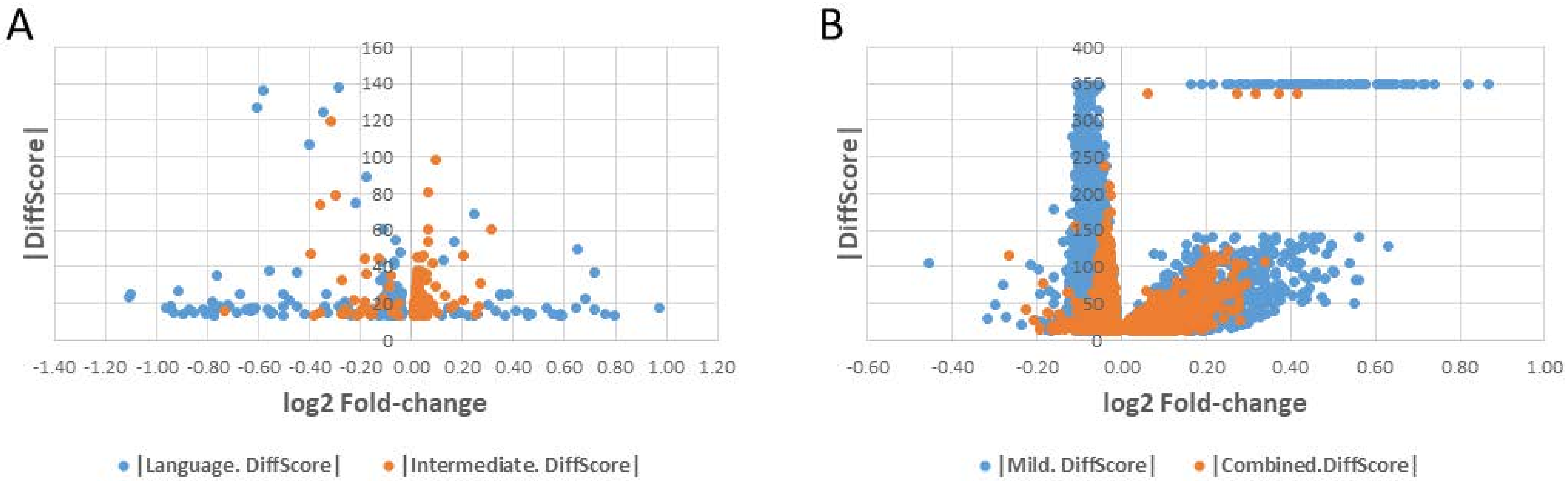

A
160
140
120
100
80
60
40
20
0
-1.40 -1.20 -1.00 -0.80 -0.60 -0.40 -0.20 0.00 0.20 0.40 0.60 0.80 1.00 1.20
log2 Fold-change
|DiffScore|
|Language. DiffScore|
|Intermediate. DiffScore|
B
400
350
300
250
200
150
100
50
0
-0.60 -0.40 -0.20 0.00 0.20 0.40 0.60 0.80 1.00
log2 Fold-change
|DiffScore|
|Mild. DiffScore|
|Combined.DiffScore|

Fig. S2

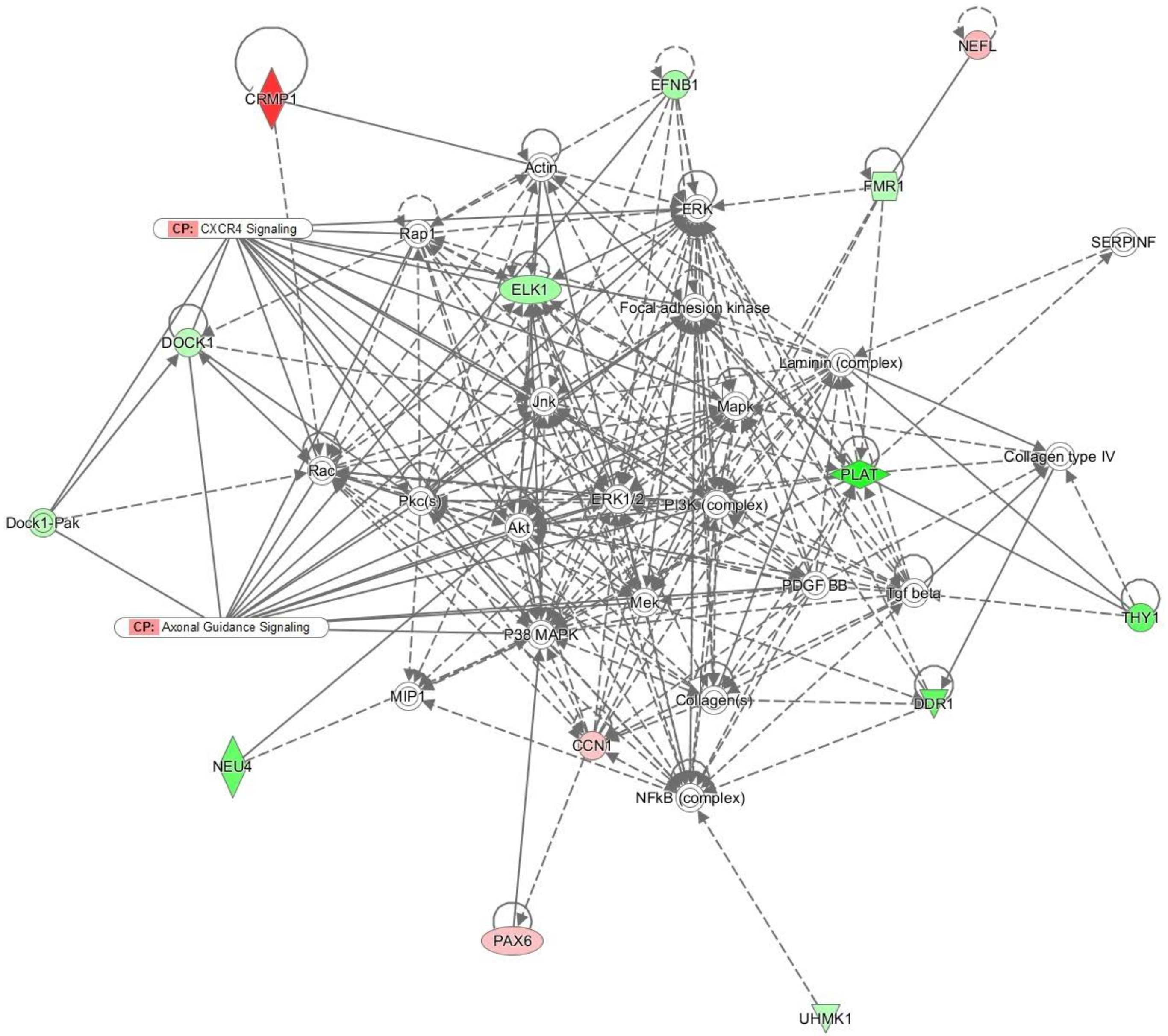
CRMP1
NEFL
EFNB1
Actin
FMR1
ERK
CP: CXCR4 Signaling
Rap1
SERPINF
ELK1
Focal adhesion kinase
DOCK1
Laminin (complex)
Jnk
Mapk
Collagen type IV
Rac
PLAT
Dock1-Pak
Pkc(s)
ERK1/2
PI3K (complex)
Akt
PDGF BB
Tgf beta
Mek
THY1
CP: Axonal Guidance Signaling
P38 MAPK
MIP1
Collagen(s)
DDR1
CCN1
NEU4
NFkB (complex)
PAX6
UHMK1

Fig. S3

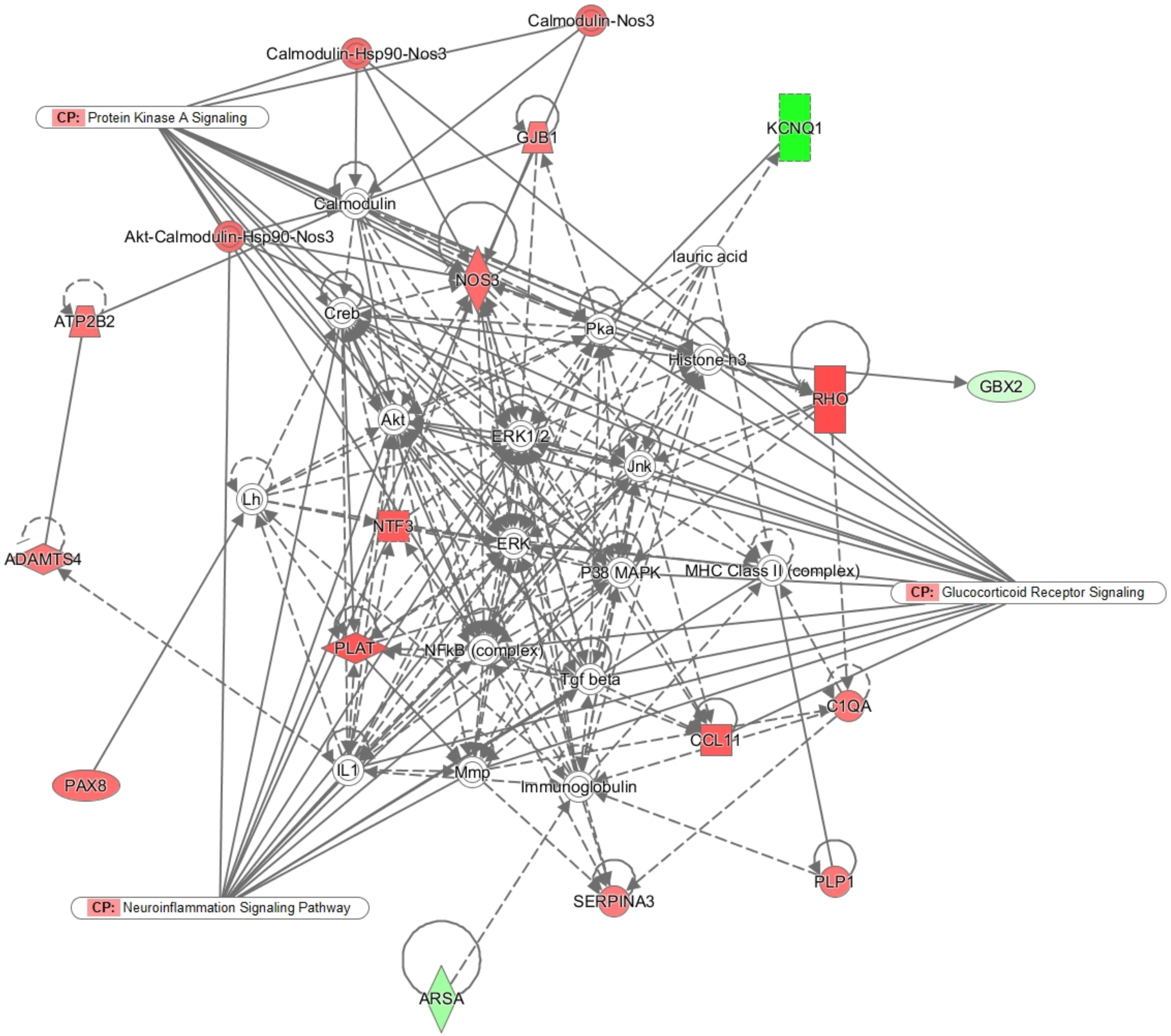
Calmodulin-Nos3
Calmodulin-Hsp90-Nos3
CP: Protein Kinase A Signaling
KCNQ1
GJB1
Calmodulin
Akt-Calmodulin-Hsp90-Nos3
lauric acid
NOS3
ATP2B2
Creb
Pka
Histone h3
GBX2
RHO
Akt
ERK1/2
Jnk
Lh
NTF3
ERK
ADAMTS4
P38 MAPK
MHC Class II (complex)
CP: Glucocorticoid Receptor Signaling
PLAT
NFkB (complex)
Tgf beta
C1QA
CCL11
IL1
Mmp
Immunoglobulin
PAX8
PLP1
SERPINA3
CP: Neuroinflammation Signaling Pathway
ARSA

Fig. S4

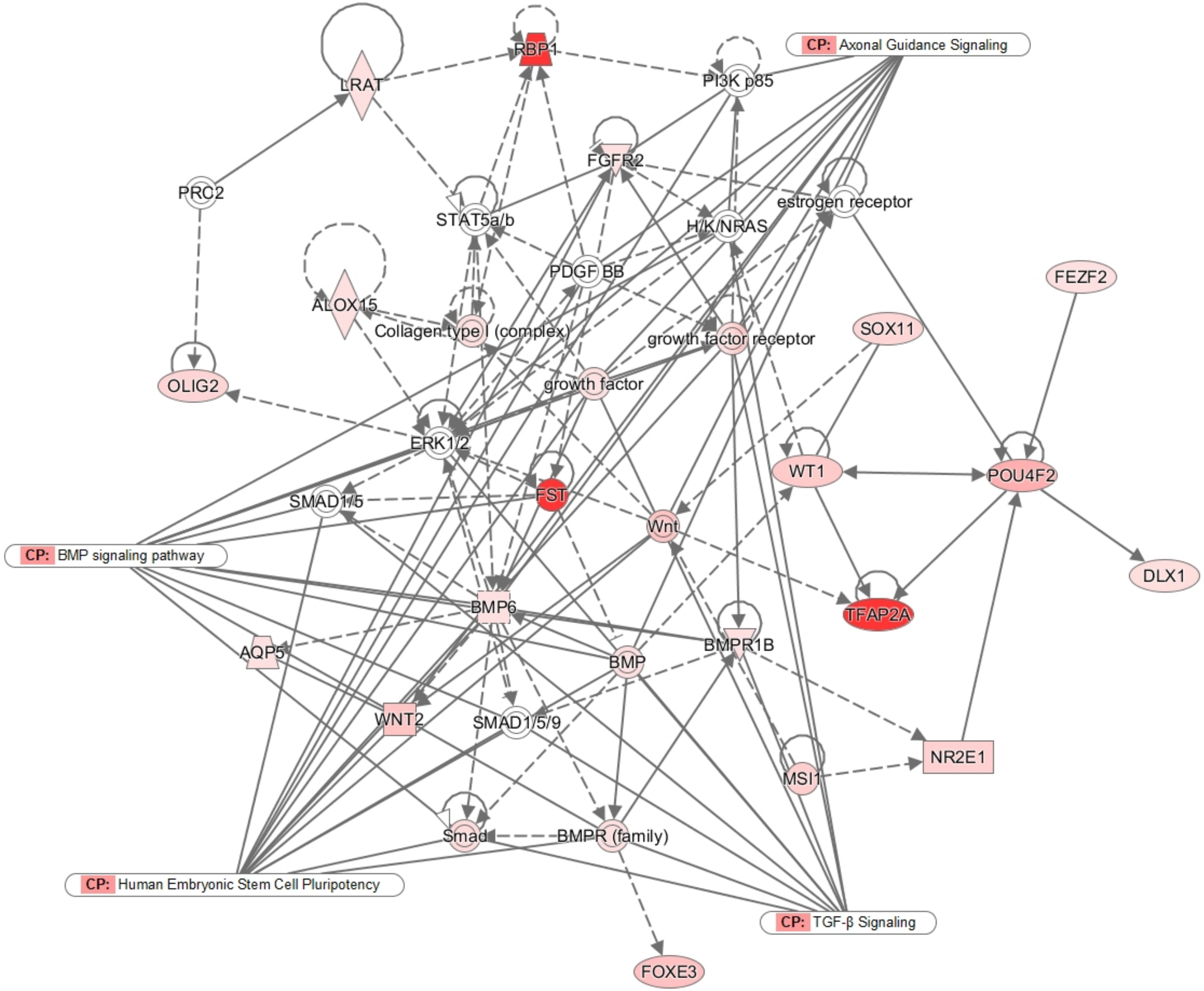

LRAT
RBP1
PI3K p85
CP: Axonal Guidance Signaling
FGFR2
PRC2
STAT5a/b
estrogen receptor
H/K/NRAS
PDGF BB
FEZF2
ALOX15
Collagen type I (complex)
growth factor receptor
SOX11
OLIG2
growth factor
ERK1/2
WT1
POU4F2
SMAD1/5
FST
Wnt
CP: BMP signaling pathway
DLX1
TFAP2A
BMP6
AQP5
BMPR1B
BMP
WNT2
SMAD1/5/9
NR2E1
MSI1
Smad
BMPR (family)
CP: Human Embryonic Stem Cell Pluripotency
CP: TGF-β Signaling
FOXE3

Fig. S5

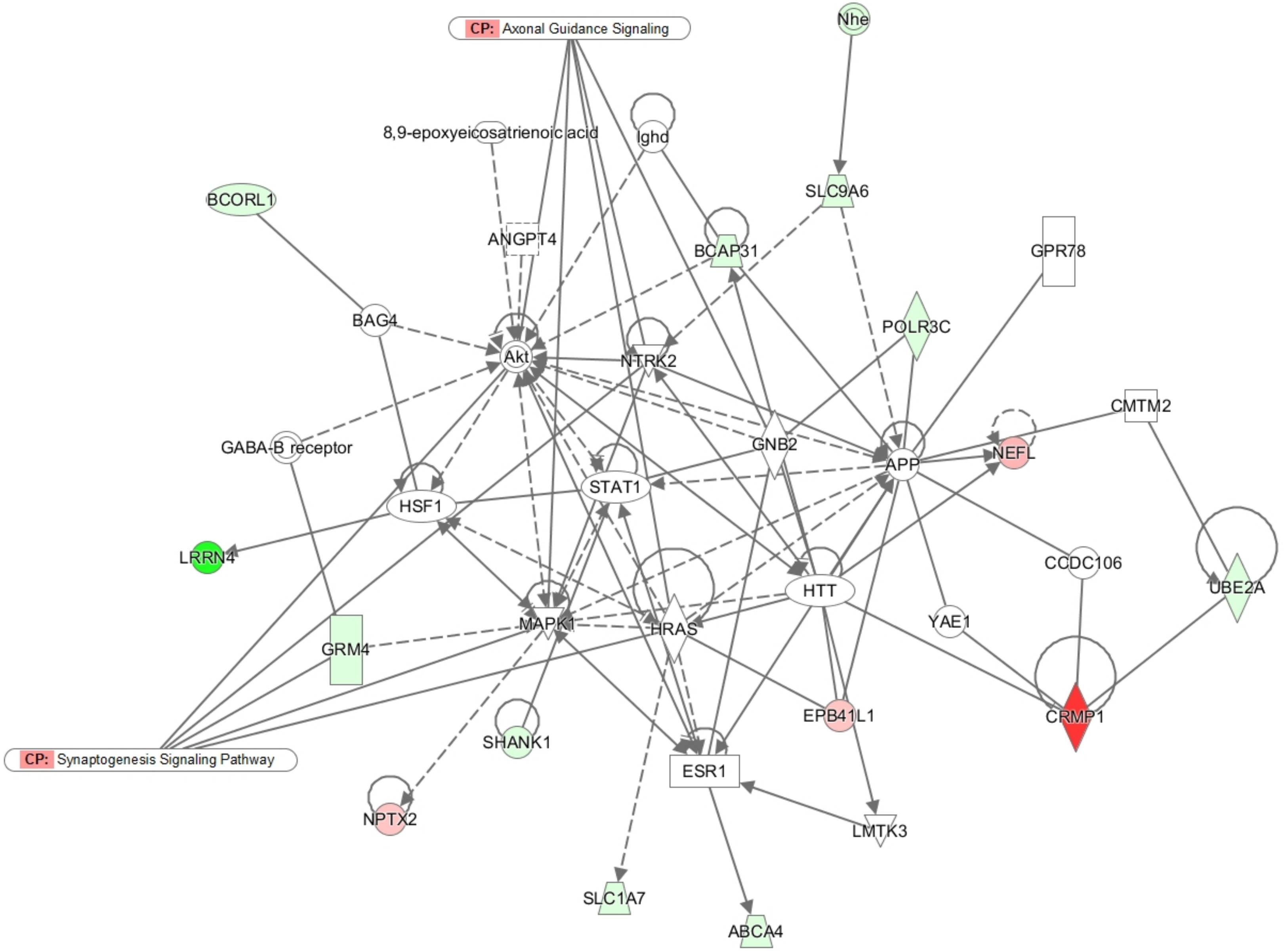
CP: Axonal Guidance Signaling
Nhe
8,9-epoxyeicosatrienoic acid
Ighd
BCORL1
SLC9A6
ANGPT4
BCAP31
GPR78
BAG4
POLR3C
Akt
NTRK2
CMTM2
GNB2
GABA-B receptor
APP
NEFL
STAT1
HSF1
LRRN4
CCDC106
HTT
UBE2A
YAE1
MAPK1
HRAS
GRM4
EPB41L1
CRMP1
SHANK1
CP: Synaptogenesis Signaling Pathway
ESR1
NPTX2
LMTK3
SLC1A7
ABCA4

Fig. S6

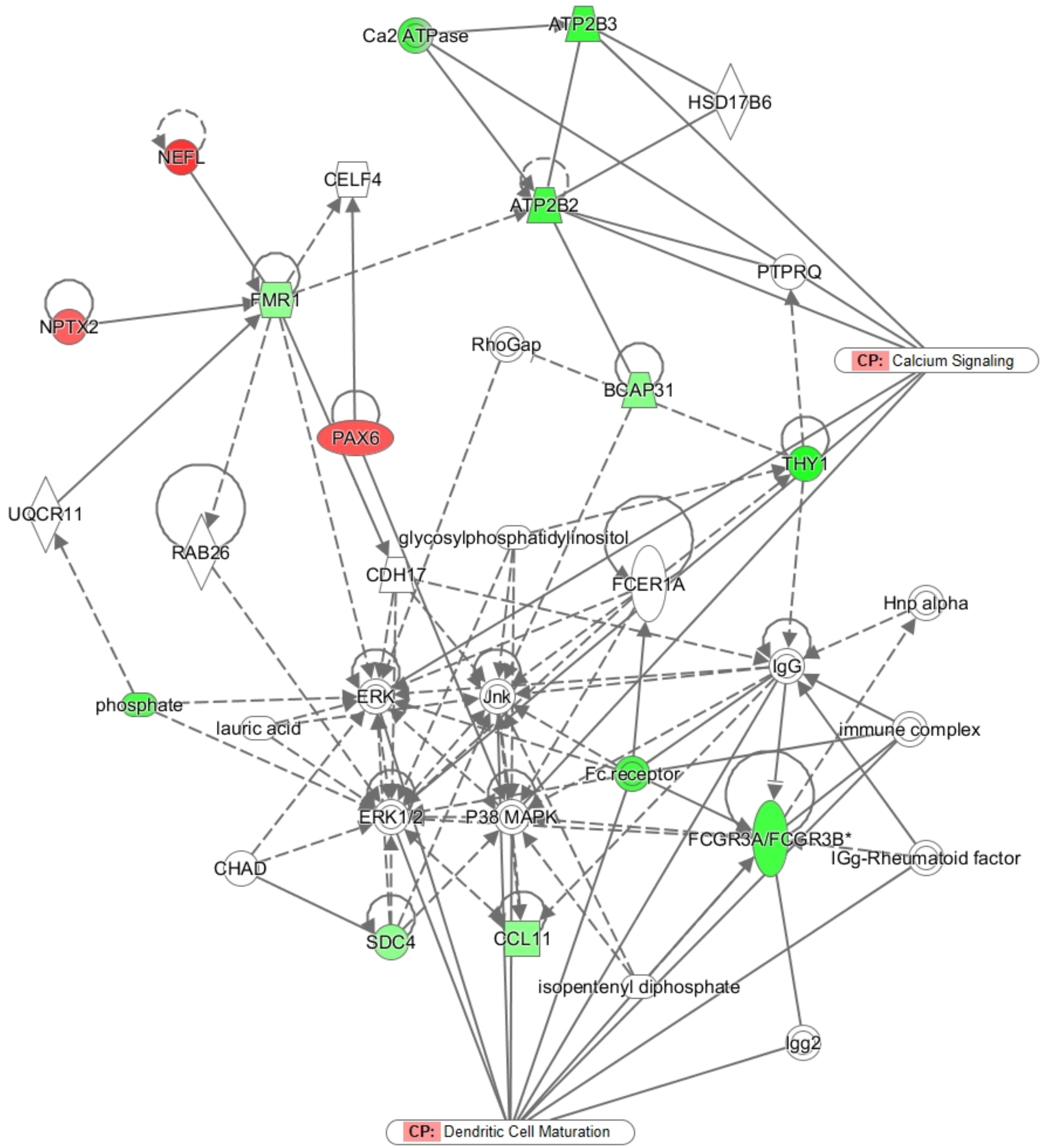
Ca2 ATPase
ATP2B3
HSD17B6
NEFL
CELF4
ATP2B2
PTPRQ
NPTX2
FMR1
RhoGap
BCAP31
CP: Calcium Signaling
PAX6
THY1
UQCR11
RAB26
glycosylphosphatidylinositol
CDH17
FCER1A
Hnp alpha
IgG
phosphate
ERK
Jnk
lauric acid
immune complex
Fc receptor
ERK1/2
P38 MAPK
FCGR3A/FCGR3B*
IGg-Rheumatoid factor
CHAD
SDC4
CCL11
isopentenyl diphosphate
Igg2
CP: Dendritic Cell Maturation

Fig. S7

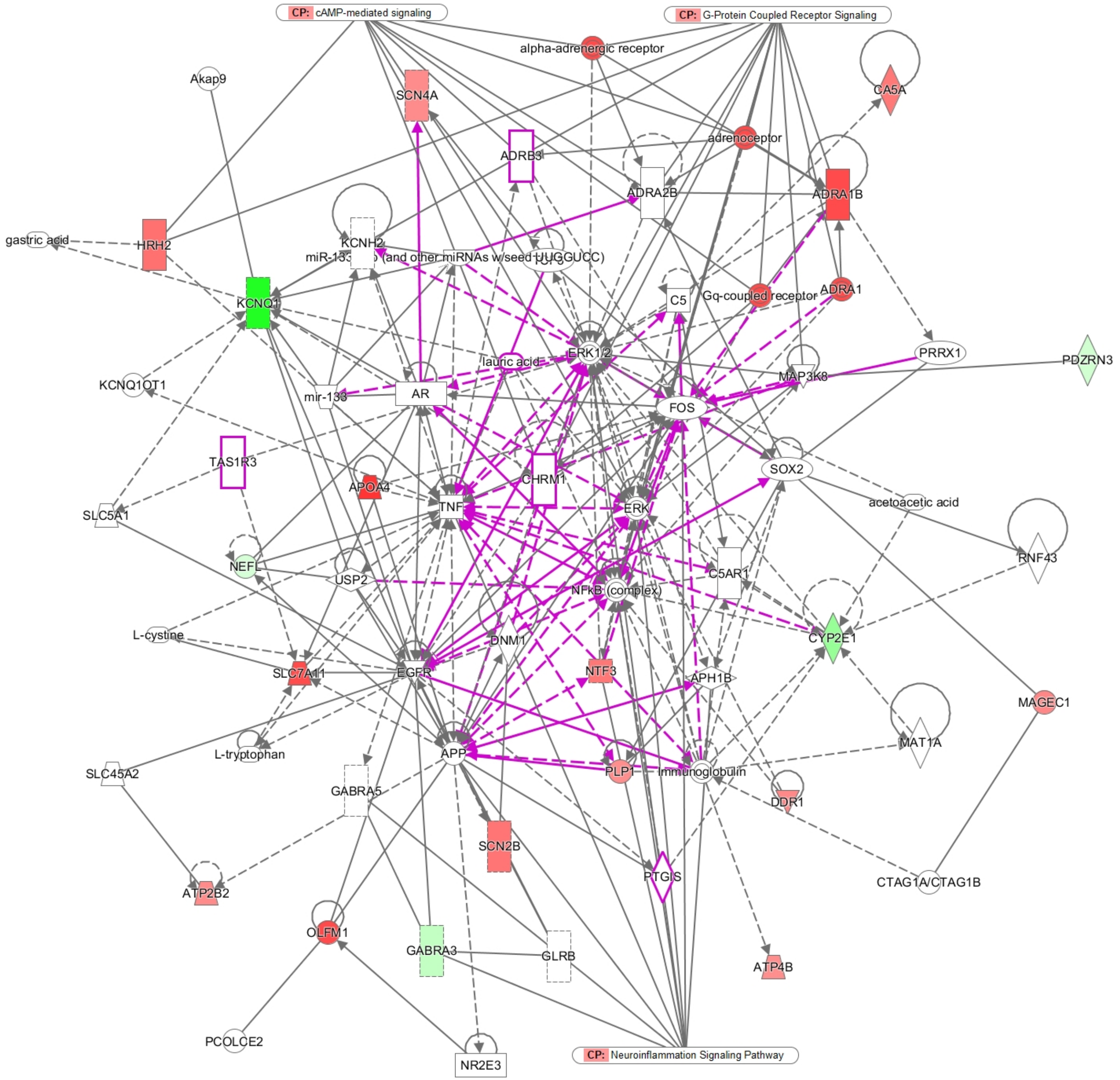
CP: cAMP-mediated signaling
CP: G-Protein Coupled Receptor Signaling
CP: Neuroinflammation Signaling Pathway
alpha-adrenergic receptor
adrenoceptor
Gq-coupled receptor
Akap9
SCN4A
CA5A
ADRB3
ADRA2B
ADRA1B
ADRA1
gastric acid
HRH2
KCNH2
miR-133 (and other miRNAs w/seed UUGGUCC)
KCNQ1
C5
ERK1/2
PRRX1
PDZRN3
KCNQ1OT1
mir-133
AR
lauric acid
MAP3K8
FOS
SOX2
TAS1R3
APOA4
CHRM1
TNF
ERK
acetoacetic acid
SLC5A1
RNF43
NEFL
USP2
NFkB (complex)
C5AR1
CYP2E1
L-cystine
DNM1
SLC7A11
EGFR
NTF3
APH1B
MAGEC1
L-tryptophan
MAT1A
SLC45A2
APP
PLP1
Immunoglobulin
GABRA5
DDR1
SCN2B
PTGIS
CTAG1A/CTAG1B
ATP2B2
OLFM1
GABRA3
GLRB
ATP4B
PCOLCE2
NR2E3

Fig. S8

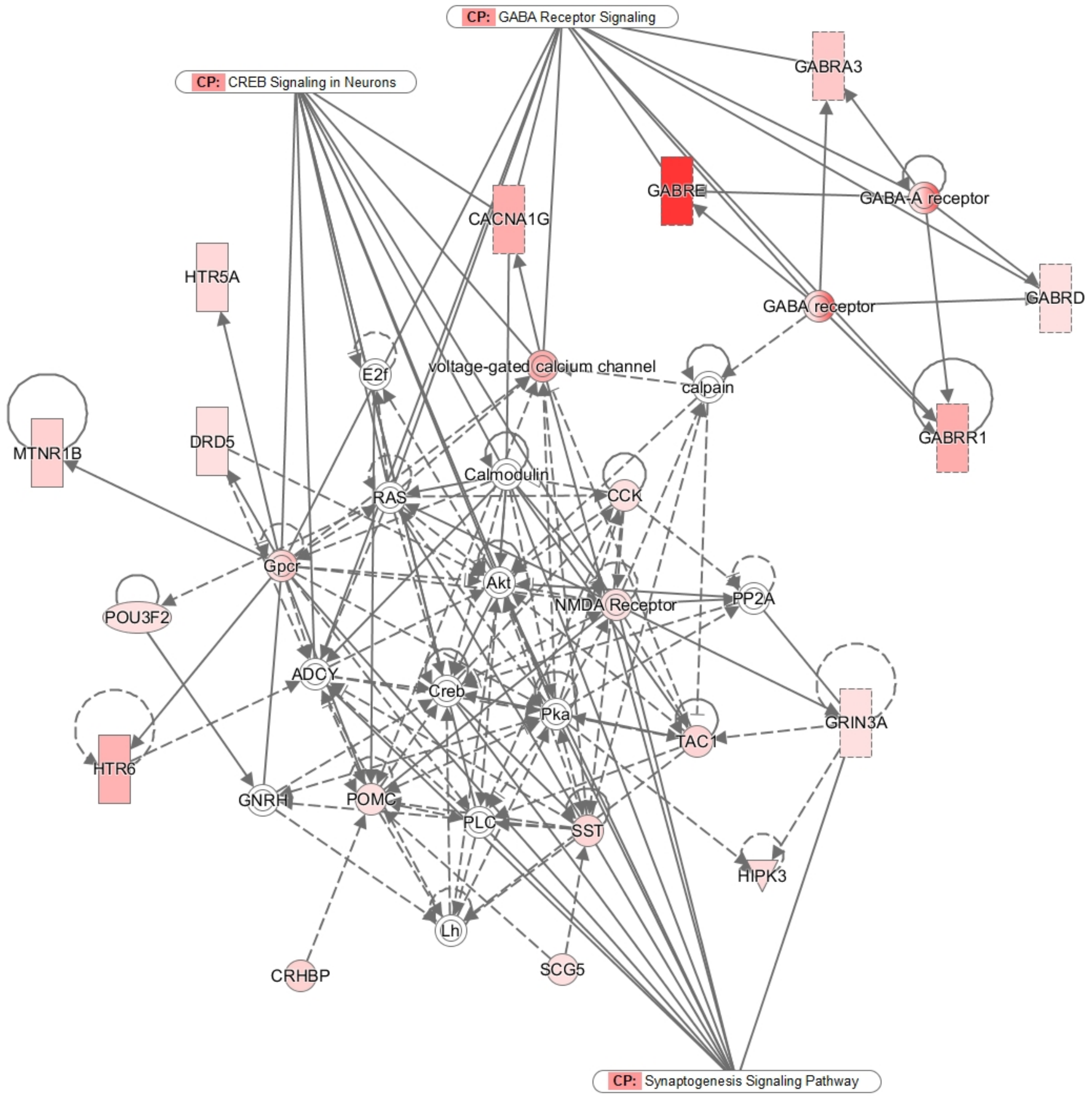
CP: GABA Receptor Signaling
CP: CREB Signaling in Neurons
GABRA3
GABRE
GABA-A receptor
CACNA1G
HTR5A
GABA receptor
GABRD
E2f
voltage-gated calcium channel
calpain
MTNR1B
DRD5
GABRR1
Calmodulin
RAS
CCK
Gpcr
Akt
PP2A
NMDA Receptor
POU3F2
ADCY
Creb
Pka
GRIN3A
TAC1
HTR6
GNRH
POMC
PLC
SST
HIPK3
Lh
CRHBP
SCG5
CP: Synaptogenesis Signaling Pathway

Fig. S9

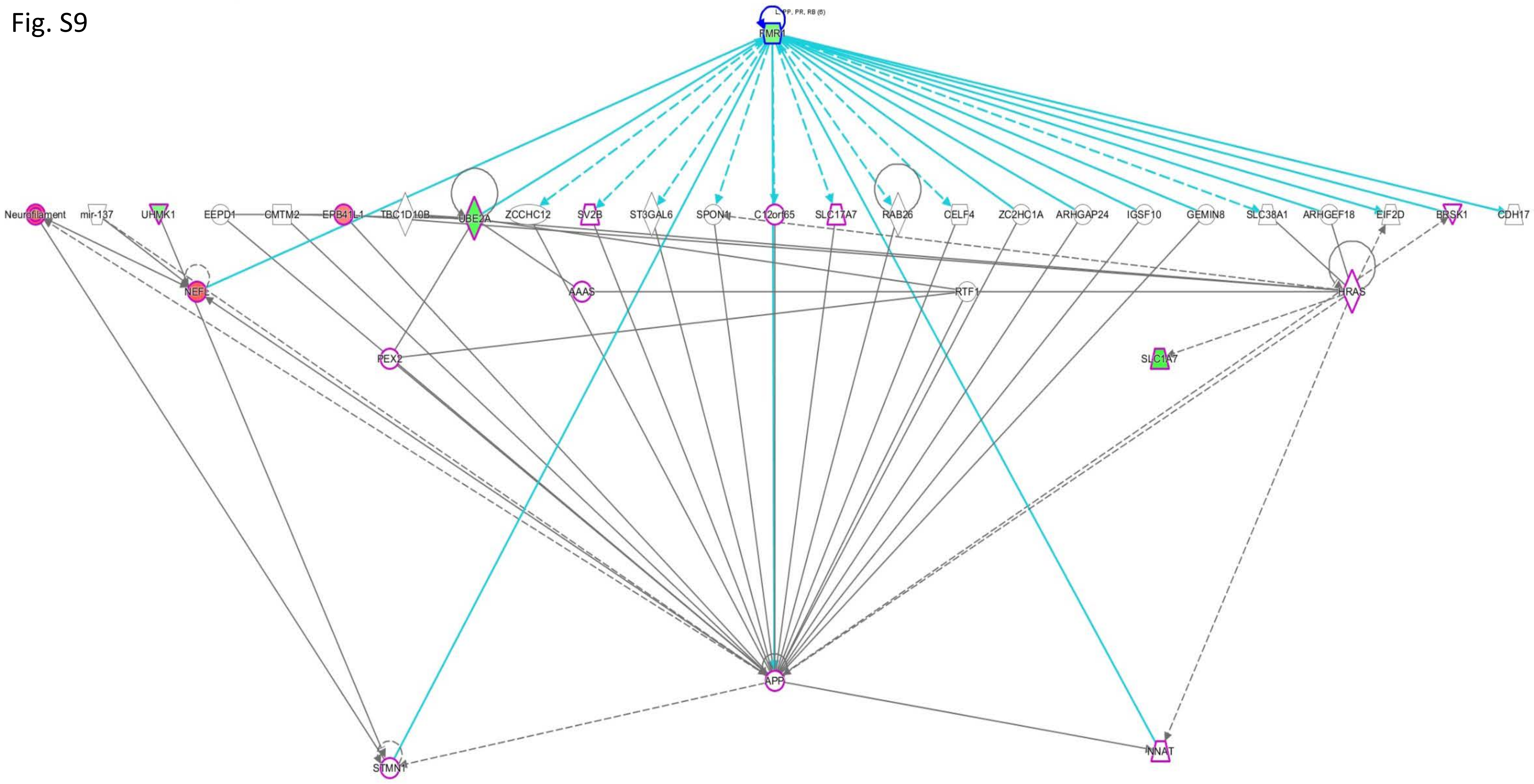
L, PP, PR, RB (6)
FMR1
Neurofilament
mir-137
UHMK1
EEPD1
CMTM2
EPB41L1
TBC1D10B
UBE2A
ZCCHC12
SV2B
ST3GAL6
SPON1
C12orf65
SLC17A7
RAB26
CELF4
ZC2HC1A
ARHGAP24
IGSF10
GEMIN8
SLC38A1
ARHGEF18
EIF2D
BRSK1
CDH17
NEFL
AAAS
RTF1
HRAS
PEX2
SLC1A7
APP
STMN1
NNAT
