# Supplementary material for: Phenotypic Subtyping and Re-Analysis of Existing Methylation Data from Autistic Probands in Simplex Families Reveal ASD Subtype-Associated Differentially Methylated Genes and Biological Functions
Source: Int J Mol Sci. 2020 Sep 19;21(18):6877. doi: 10.3390/ijms21186877 (PMC7555936; doi:10.3390/ijms21186877)
Supplement: Supplementary file 1 [file ijms-21-06877-s001.zip › ijms-927106-supplementary/Suppl. Materials_revised MS/Table S8. Demographic information for individuals in study_EDIT.pdf]

	Mild		Intermediate		Severely Language-Impaired		Combined	
	Probands	Siblings	Probands	Siblings	Probands	Siblings	Probands	Siblings
Male gender, *N*	149	149	121	121	22	22	292	292
Age at time of ADI-R: mean ± SD (range), years*	1.96 ± 1.24 (0.17–7)	—	1.76 ± 1.22 (0.08–9)	—	1.49 ± 0.65 (0.75–3)	—	1.84 ± 1.20 (0.08–9)	—
Age at time of sampling: mean ± SD (range), years[a]	9.10 ± 3.38 (4–17.75)	9.41 ± 3.93 (3–20.83)	10.57 ± 3.36 (4.08–17.67)	11.59 ± 4.74 (4–24.58)	8.25 ± 3.71 (4.25–16.17)	10.37 ± 4.30 (4.08–19.33)	9.65 ± 3.48 (4–17.75)	10.38 ± 4.42 (3–24.58)
Race/ethnicity (%*N)*								
White	106 (71.14%)	106 (71.14%)	86 (71.07%)	86 (71.07%)	14 (63.64%)	14 (63.64%)	206 (70.55%)	206 (70.55%)
Black or African American	2 (1.34%)	2 (1.34%)	3 (2.48%)	3 (2.48%)	0 (0%)	0 (0%)	5 (1.71%)	5 (1.71%)
Asian	4 (2.68%)	4 (2.68%)	2 (1.65%)	2 (1.65%)	2 (9.09%)	2 (9.09%)	8 (2.74%)	8 (2.74%)
Hawaiian or Pacific Islander	0 (0%)	0 (0%)	1 (0.83%)	1 (0.83%)	0 (0%)	0 (0%)	1 (0.34%)	1 (0.34%)
More than one race	8 (5.37%)	8 (5.37%)	8 (6.61%)	8 (6.61%)	2 (9.09%)	2 (9.09%)	18 (6.16%)	18 (6.16%)
Unknown or not specified	29 (19.46%)	29 (19.46%)	21 (17.36%)	21 (17.36%)	4 (18.18%)	4 (18.18%)	54 (18.49%)	54 (18.49%)

SD standard deviation

* Age of proband at the time of administration and scoring of the Autism Diagnostic Interview-Revised (ADI-R). Ages unknown for 2/149 probands in the mild subgroup and 2/121 probands in the intermediate subgroup.

[a] Age of individual at the time blood samples were collected for the Simons Simplex Collection (SSC) repository.
